# Brain-inspired computing with fluidic iontronic nanochannels

**DOI:** 10.1073/pnas.2320242121

**Published:** 2024-04-24

**Authors:** Tim M. Kamsma, Jaehyun Kim, Kyungjun Kim, Willem Q. Boon, Cristian Spitoni, Jungyul Park, René van Roij

**Affiliations:** ^a^Institute for Theoretical Physics, Department of Physics, Utrecht University, Utrecht 3584, The Netherlands; ^b^Mathematical Institute, Department of Mathematics, Utrecht University, Utrecht 3584, The Netherlands; ^c^Department of Mechanical Engineering, Sogang University, Seoul 04107, Republic of Korea

**Keywords:** iontronics, neuromorphics, memristor, reservoir computing, nanofluidics

## Abstract

The brain’s remarkable and efficient information processing capability is driving research into brain-inspired (neuromorphic) computing paradigms. Artificial aqueous ion channels are emerging as an exciting platform for neuromorphic computing, representing a departure from conventional solid-state devices by directly mimicking the brain’s fluidic ion transport. Supported by a quantitative theoretical model, we present easy-to-fabricate tapered microchannels that embed a conducting network of fluidic nanochannels between a colloidal structure. Due to transient salt concentration polarization, our devices are volatile memristors (memory resistors) that are remarkably stable. The voltage-driven net salt flux and accumulation, that underpin the concentration polarization, surprisingly combine into a diffusionlike quadratic dependence of the memory retention time on the channel length, allowing channel design for a specific timescale. We implement our device as a synaptic element for neuromorphic reservoir computing. Individual channels distinguish various time series, that together represent (handwritten) numbers, for subsequent in silico classification with a simple readout function. Our results represent a significant step toward realizing the promise of fluidic ion channels as a platform to emulate the rich aqueous dynamics of the brain.

## Significance

The brain’s computing principles (neurons connected by synapses) and information carriers (ions in water) both differ fundamentally from those of conventional computers. Building on this distinction, we present an aqueous memristor that emulates the brain’s short-term synaptic plasticity features through ion transport in water, mirroring the natural processes in the brain. This device, which is inspired by and understood through a theoretical model, is applied as a synaptic element for reservoir computing, a brain-inspired machine learning framework. Thus we implement a brain-inspired computing element in a brain-inspired fluidic medium, representing a considerable step toward computing devices that proverbially both walk and talk like the brain.

Neuromorphic computing aims to replicate the information processing of the human brain, which is orders of magnitude more energy efficient than conventional computing devices (1, 2). This is paramount as the unsustainable trend of energy consumption by computers is growing at an exponential rate, driving investigations into brain-inspired computing paradigms (3). To pursue brain-like information processing, a device structure that goes beyond the conventional von Neumann architecture is necessary (4). To this end, memristors (memory resistors) have emerged as promising artificial analogues to biological synapses that enable brain-inspired circuit architectures (2, 3, 5–7).

Despite the successful implementation of memristors in various conventional platforms, the vast majority of these devices consist (at least partially) of solid-state components, rely on only a single information carrier (usually electrons or holes), and only respond to electric driving forces (3, 7). These limitations contrast with the brain’s nimble synapses, which can utilize both electrical and chemical signals by relying on transport in an aqueous environment of various ionic and molecular species in parallel (8). In light of this disparity, an emerging and exciting approach seeks inspiration not only from the architecture of the brain, but also from its aqueous medium and ionic signal carriers (9). These so-called iontronic devices employ ions moving in an aqueous environment to carry information, offering the promise of multiple information carriers, chemical regulation, and biointegrability (10). Consequently, various iontronic memristors have been presented (11–14) that can exhibit synaptic plasticity features (15, 16), and utilize chemical regulation (17, 18). Additionally, recent advancements have been made in employing iontronic devices for signaling and computing, with theoretical proposals (19–21), and demonstrations of traditional truth tables (22–24), respectively. Despite these prospects, the development of aqueous neuromorphic devices is still in its infancy and neuromorphic computing implementations remain a challenge (10, 25, 26).

Here, we theoretically propose and experimentally realize the implementation of an aqueous volatile memristor as a synaptic element for iontronic neuromorphic computing. This device is stable over long periods of time, providing reliable and distinct responses to temporal inputs, enabling its use as computing element. Additionally, device fabrication is fast, cost-effective, and easy via a soft-lithography process that is almost free-shaping. By constructing a channel of a certain chosen length, made easy by the flexible fabrication process, we can design our channel to feature a specific timescale chosen from a wide range, a desirable property of memristive devices (27). The volatile nature, i.e., decaying conductance memory when driving forces are removed, with adjustable memory retention times makes our memristor a promising candidate for reservoir computing, a brain-inspired machine learning framework which has drawn attention due to its capability of handling complex time series and sequential tasks (28–33). We implement benchmark protocols of classifying (handwritten) numbers that are encoded as temporal signals. Our aqueous channels process the time series, distinguishing them for subsequent in silico classification with a simple readout function, performing (at least) comparable with more conventional solid-state platforms employing similar protocols (32–35).

Our iontronic device is understood through a quantitative theoretical model that directly derives from continuum transport equations and identifies an inhomogeneous ionic space charge density, observed between the colloids (36), as (a general) main ingredient to induce salt concentration polarization and consequent ion current rectification (ICR). Moreover, our theory elucidates how the voltage-driven net salt flux and accumulation, that underpin the (transient) concentration polarization, surprisingly combine into a diffusionlike conductance memory timescale that quadratically depends on the channel length. Consequently, the theory correctly predicts the voltage-dependent (dynamic) conductance, thereby facilitating a great acceleration of the experiments by pointing out the relevant signal voltages, signal timescales, and suitable reservoir computing protocol.

The combination of i) a stable fluidic memristor that can be designed to feature a specific memory timescale, made with ii) an almost free-shaping and cost-effective soft-lithography fabrication process, iii) a theoretical model that quantitatively describes and predicts the device dynamics, and iv) the implementation of an aqueous iontronic device as an element for neuromorphic computing, forms a significant advancement toward developing iontronic devices that can facilitate the wealth of communication pathways harnessed by the brain.

## 1. Fluidic NCNM Memristor: Theory and Experiment

**Fig. 1.**
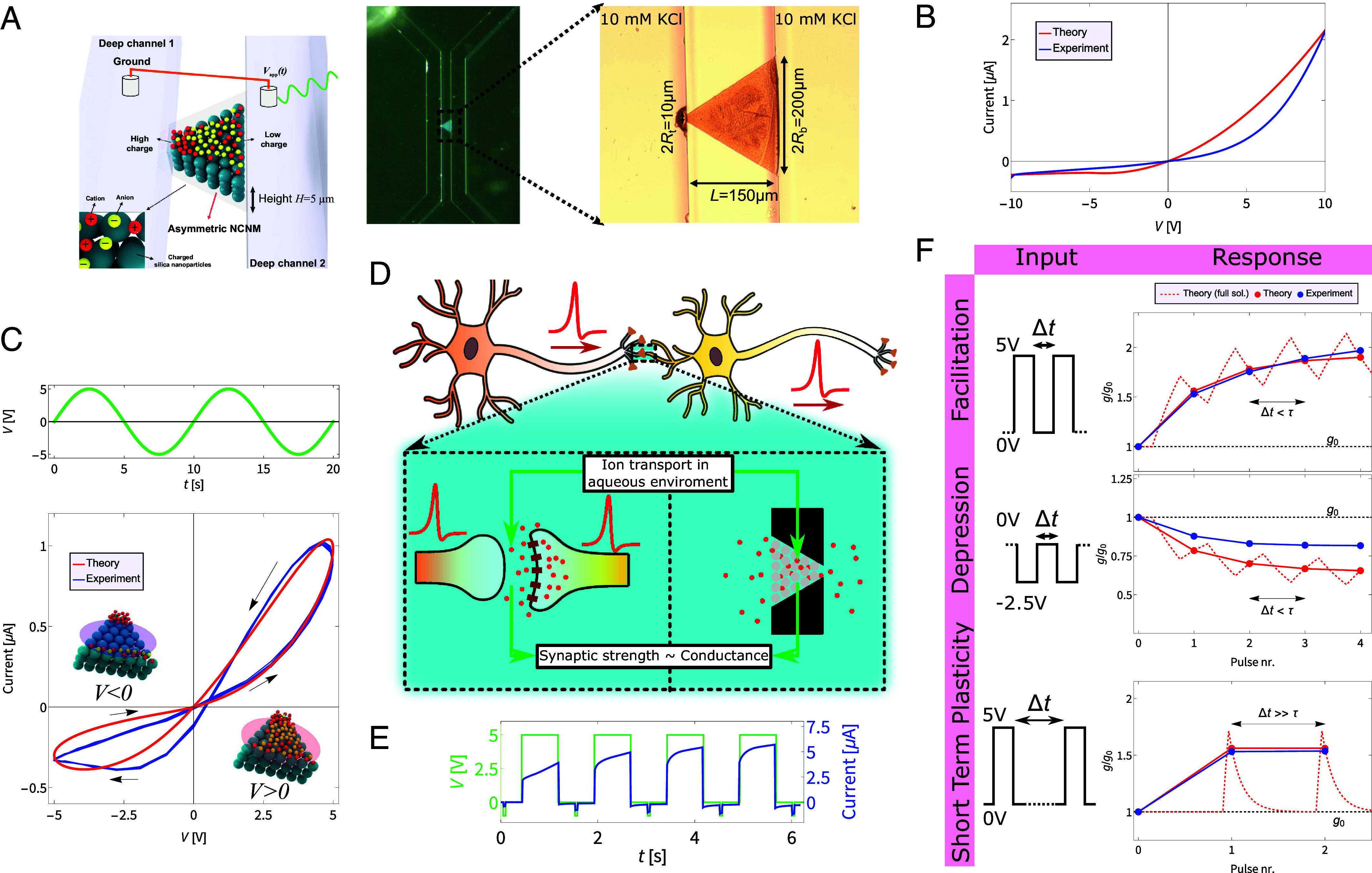
Features and properties of our iontronic memristor through theory and experiment. (*A*) Schematic (*Left*) and pictures (*Right*) of the device. The channel connects two reservoirs of aqueous KCl electrolyte and incorporates a rigid colloidal structure, forming a network of nanochannels between the colloids. (*B*) Steady-state *I*–*V* curve observed in experiments (blue) and predicted by our theory (red), showing a similar current rectification property. (*C*) Dynamic *I*–*V* curve in response to a sinusoidal voltage over the channel (*Top*, green). The theory (*Bottom*, red) and the experiments (bottom, blue) both exhibit a similar pinched hysteresis loop. (*D*) Simplified schematic of synaptic signal transmission. An action potential triggers neurotransmitter release (not depicted) from the presynaptic neuron (orange), binding to receptors on the postsynaptic neuron (yellow), potentially inducing ion transport and altering its membrane potential (8). The dynamic channel conductance is analogous to the synaptic strength. (*E*) Current measurements (blue) when four consecutive 5 V pulses and five read pulses (green) are applied. (*F*) Short-term plasticity (STP) features observed in the channel (blue) and predicted by the theory, where we show the full (numerical) solution for $g\left(t\right)/g_{0}$ (red, dashed) and the measurements this would correspond to in the experiment (red, dots). Four consecutive voltage pulses with Δt smaller than the channel’s memory retention time τ leads to facilitation (*Top*) and depression (*Middle*) for pulses of 5V and −2.5V, respectively. The short-term characteristic is clearly visible when Δt≫τ, in this instance no cumulative change in conductance is found (*Bottom*).

Our experimental system, as shown in Fig. 1*A*, consists of a tapered microfluidic channel of uniform height H=5μm and a width that linearly decreases over its length L=150μm from $2R_{b}=200\;\mu$m at the broad base to $2R_{t}=10\;\mu$m at the narrow tip. The channel, which connects two deep reservoirs containing an aqueous 10 mM KCl electrolyte, is filled with a rigid face-centered cubic (fcc) crystal structure at a near-close-packed volume fraction η≃0.74 of charged silica spheres with radius a=100 nm and approximate surface charge density $\sigma_{c}=-0.01$ Cm^−2^. To form the colloidal structure, the colloids, dispersed in a 70% ethanol solution, are injected into deep channel 1 (as in Fig. 1*A*) of 100μm height, filling the tapered shallow channel up to the base through capillary action. The fluid halts at the interface between the shallow channel and deep channel 2 due to the Laplace pressure and consequently evaporates, promoting nanoparticle self-assembly into a close-packed fcc. The device is then dried and prepared for the experiments by filling it with the aqueous electrolyte. The electrolyte in the space between the colloids forms a conducting nanochannel network membrane (NCNM) with pore spaces up to tens of nm in the tetrahedral and octahedral holes of the fcc lattice. This connected porous structure can support an ionic current I driven by a voltage drop V over the length of the channel, defined as tip minus base voltage (i.e., $V=-V_{\mathrm{app}}$). More system parameters and characteristics are laid out in *SI Appendix*.

Some of us previously showed that these types of colloid-filled microchannels are ionic diodes with excellent ICR ratios of up to 55 for the channels on which the devices in this work are based (36), and even up to ∼1,600, the highest reported value at the time, for a more recent version containing two colloidal structures of opposite surface charge (37). The physical phenomenon that underpins the ICR is the strong voltage-dependent salt concentration polarization, hypothesized to be induced by an experimentally observed inhomogeneous ionic space charge density between the colloidal particles (36). Calculations on a standard colloidal Wigner-Seitz cell model, presented in detail in *SI Appendix*, show that a small (essentially invisible) variation of order ∼1% in the colloidal packing fraction between base and tip of the channel can alter the colloidal surface charge density by several 10% for a fixed zeta potential. By assuming macroscopic electroneutrality (38, 39), the ionic space charge in the (thin) electric double layers of the charged colloids varies similarly, thereby providing a natural explanation for the hitherto unexplained ionic charge density profile. In order to theoretically investigate the hypothesis that the inhomogeneous space charge density is responsible for the ICR, we employ standard Poisson–Nernst–Planck (PNP) equations for ionic transport to explain that the inhomogeneous ionic space charge density indeed leads to current rectification. Our theoretical framework, based on an efficient slab-averaging approach in tapered channel geometries (40, 41), is described below, while the detailed calculations can be found in *SI Appendix*.

The PNP equations form an effective theoretical framework to analyze ion transport in charged porous materials (42). However, the complex three-dimensional geometric structure of the NCNM, with features on length scales varying from the colloidal surface-surface distance all the way up to the channel length, introduces intricate numerical challenges for fully spatially resolved solutions of the PNP equations. To simplify, we consider slab-averages, i.e., the average along a cross-section (38, 40, 41, 43, 44), of the electric potential and the ionic concentrations in the porous structure between the colloids. Although this sacrifices on nanoscale details, it does account for the pinched electric field lines toward the channel tip and for the spatial variation of the ionic charge density. Through this method we reduce the three-dimensional Nernst–Planck equation to a one-dimensional form, providing an expression for the total salt and charge flux through the channel. The divergence of the total salt flux qualitatively shows that the experimentally observed inhomogeneous ionic space charge density forms a source (sink) term of salt, resulting in salt accumulation (depletion) upon a positive (negative) applied voltage V. Quantitatively, a divergence-free steady-state condition on the total salt flux provides a differential equation for the voltage-dependent slab-averaged salt concentration profile, which we solve analytically. By viewing the channel as a series of conductive slabs, with the conductance of each slab proportional to the (now known) voltage-dependent salt concentration, we calculate the steady-state channel conductance $g_{\infty }\left(V\right)=I\left(V\right)/V$. This describes how an increase (decrease) in salt in the channel at positive (negative) voltages makes the channel more (less) conductive. Our theory thus quantitatively confirms the experimental hypothesis that the ionic space charge distribution results in salt concentration polarization and hence in current rectification (36). Moreover, leveraging the general analytical nature of our theory, we demonstrate that any inhomogeneous ionic space charge density in generic channels (provided they are well described by slab-averaged PNP equations) is the key ingredient for a source-sink term of salt and thus for current rectification, derived in detail in *SI Appendix*. Therefore we not only provide a mechanistic insight as to how the space charge leads to current rectification in the channel of present interest, but this understanding could also explain current rectification in channels with other sources of space charge densities and with other geometries (23, 37). Furthermore, this insight may provide inspiration for future design of devices that exhibit current rectification.

In Fig. 1*B* we plot the predicted steady-state current (red) and the experimentally observed current (blue), revealing a similar current rectification. The experimentally observed ICR ratio of 11 is lower than one of our earlier (slightly more complicated) microchannels (36); however, it is sufficient for this work and we believe it will be straightforward to optimize our channel for higher ratios in the future as higher ratios were already achieved in similar channels (36, 37).

Up to this point, we treated the system in steady state. When extending our view to the device dynamics we need to consider the time it takes for ions to accumulate into or deplete out of the channel. Utilizing our aforementioned expression for the total salt flux through the channel $V^{\prime }$, we calculate the net flux $\gamma V^{\prime }$ into the channel upon a small applied voltage and find (see *SI Appendix* for details) that γ∝D/L, with L the channel length and D the ionic diffusion coefficient. The contributions to the net flux solely come from the conductive, i.e., voltage-driven, flux term in the Nernst–Planck equation. The proportionality to D/L is intuitive as the electric field strength in the channels is proportional to 1/L and all flux terms are proportional to the ionic mobilities and hence to D. With our expression for the slab-averaged salt concentration profile, we also calculate the total change in salt $\alpha V^{\prime }$ upon applying the small voltage $V^{\prime }$, and we find α∝L. This proportionality to L again is intuitive, as the volume of the channel scales with L. The ratio α/γ between this total change in salt and net flux provides an estimate for the concentration polarization timescale, given by

[1] $$\begin{array}{c} \tau =\frac{L^{2}}{4D}\xi , \end{array}$$

where ξ≈0.42 is an involved dimensionless number that depends on the ratio of the channel widths $R_{t}/R_{b}$ and on the ratio of the internal space charge density at the tip and the base (full expression in *SI Appendix*). Eq. **1** shows that τ is a diffusionlike time, which is remarkable as no diffusion terms from the Nernst–Planck equation go directly into the derivation of Eq. **1**. Nevertheless, from the aforementioned intuitive dependencies on D/L and L of the net salt flux and total change in salt, respectively, we surprisingly do retrieve a diffusionlike timescale from a voltage-driven process. Inserting our present system parameters, including $D=1.45\;\mu \;{\text{m}}^{2}\;\;{\text{ms}}^{-1}$, yields a memory timescale of τ=1.62 s.

With the steady-state conductance $g_{\infty }\left(V\right)$ and the conductance memory timescale τ, we formulate our theory for the time-dependent channel conductance g(t) resulting from a time-dependent voltage V(t). We intuitively expect g(t) to relax toward the instantaneous static conductance $g_{\infty }\left(V\left(t\right)\right)$ on a time scale τ, with a vanishing rate of change dg(t)/dt once $g\left(t\right)=g_{\infty }\left(V\left(t\right)\right)$. We employ a simple equation of motion (which we more rigorously support in *SI Appendix*) that characterizes this relaxation process and write

[2] $$\begin{array}{cc} \frac{dg(t)}{dt} & =\frac{g_{\infty }\left(V\left(t\right)\right)-g\left(t\right)}{\tau }; \end{array}$$

[3] $$\begin{array}{cc} I(t) & =g(t)V(t), \end{array}$$

where Eq. **2** is a form that was also successfully applied in previous studies on various memristors (18, 20, 41, 45) and Eq. **3** is Ohm’s law.

To demonstrate the memristive properties of our device we impose a sinusoidal voltage V(t) of amplitude 5 V and frequency f=0.1 Hz as shown in Fig. 1*C*, green. The resulting current–voltage (*I*–*V*) diagram is shown in Fig. 1*C*, blue, where a clear pinched hysteresis loop is observed, the hallmark of a memristor (46). This loop features a pronounced memory effect (i.e., more open hysteresis loop) compared to various fluidic memristive devices (12, 17, 18, 47), allowing for a wide range of comparatively high conductances. Moreover, in Fig. 1*C*, red, we see that our theory shows good agreement with experimental findings.

Memristors are recognized as artificial analogues to synapses, the connections between neurons (6). In neuronal communication, the change in membrane potential of a postsynaptic neuron, triggered by an influx of ions in response to a signal from a connected presynaptic neuron, is a measure for the synaptic connection strength (8). Similarly, our device’s measured ion current, also a result of ion flux through a membrane, draws a parallel between the channel’s conductance and synaptic strength in neurons, as schematically depicted in Fig. 1*D*. A crucial aspect of neuronal functioning is STP, which allows neurons to adjust their synaptic strength in response to recent input history, being of key importance in information processing (48). STP involves changes in the synaptic strength that decay over timescales ranging from milliseconds to minutes, where an increase of the synaptic strength is called (short-term) facilitation and a decrease (short-term) depression (48–50). To demonstrate that our fluidic memristor can mimic these aspects of neuronal STP, we apply four consecutive positive and negative “write-pulses” of 5V and −2.5V, respectively, with a 0.75s duration, separated by intervals of Δt=0.75s<τ smaller than the memory retention time τ. The asymmetric 5 V and −2.5 V voltages helped optimize the conductance response. We measure the channel conductance by applying small and short “read-pulses” of −1 V and duration 50 ms after each write-pulse. In Fig. 1*E* we show the current measurements (blue) when four 5 V write-pulses and five −1 V read-pulses (green) are applied, with energy consumption of ∼1–10μJ for the write-pulses and ∼10–100 nJ for the read-pulses. The read-pulses are converted to the measured channel conductances shown in Fig. 1*F*. As illustrated in Fig. 1*F*, blue, our fluidic memristor exhibits both facilitation (top graph) and depression (middle graph), hence replicating the characteristic features of neuronal STP. These results are the average of three devices with two measurements per device, each showing quantitatively similar behavior. In Fig. 1*F* (bottom graph) the short-term character of the response is prominently visible when the interval between the pulses is much longer than the typical memory retention time τ and no cumulative change in conductance is observed. Our experimental findings are mostly in good agreement with Eq. **2** shown in Fig. 1*F*, red, the only notable discrepancy being that the measured strength of depression is weaker than predicted. The overall agreement emphasizes the robustness and predictive power of the theoretical model in quantitatively characterizing the device properties.

**Fig. 2.**
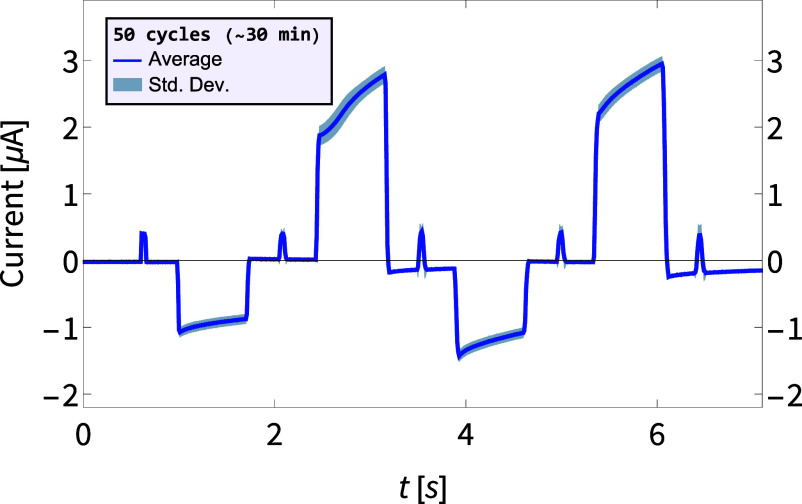
Stability of our NCNM memristors. Averaged current (blue) measured over 50 subsequent voltage pulse train cycles, taking roughly 30 min. Each train consists of four write-pulses, of −2 V and 5 V, respectively, interspersed with five read-pulses of 1 V. All cycles showed essentially the same behavior, producing a spread with SD for each current measurement around the average of maximally ∼7% (blue-gray), demonstrating the device stability.

To reliably perform neuromorphic reservoir computing our devices need to repeatedly produce the same response to signals. In Fig. 2, we demonstrate the stability and reproducibility of our device by applying 50 subsequent cycles of four write-pulses of −2 V and 5 V, respectively, taking roughly 30 min to complete. The resulting current, averaged over all 50 cycles (blue), shows a narrow spread with a SD (blue-gray) of at most ∼7%. In *SI Appendix*, we present additional measurements, including 26 cycles over 4 h for all 16 different four-pulse voltage trains, reliably finding a similar conductance modulation each cycle with conductance SD of typically a few % and no more than ∼10%. As fabrication is fast and easy, we normally constructed new devices for new sets of experiments. However, devices were in fact stable enough to be reusable, but did dry out if kept for long with our current way of storing, requiring cleaning the salt residue before using again. The device stability is an important feature that enables it to reliably distinguish a large number of different time series, underpinning the reservoir computing, as we discuss later.

**Fig. 3.**
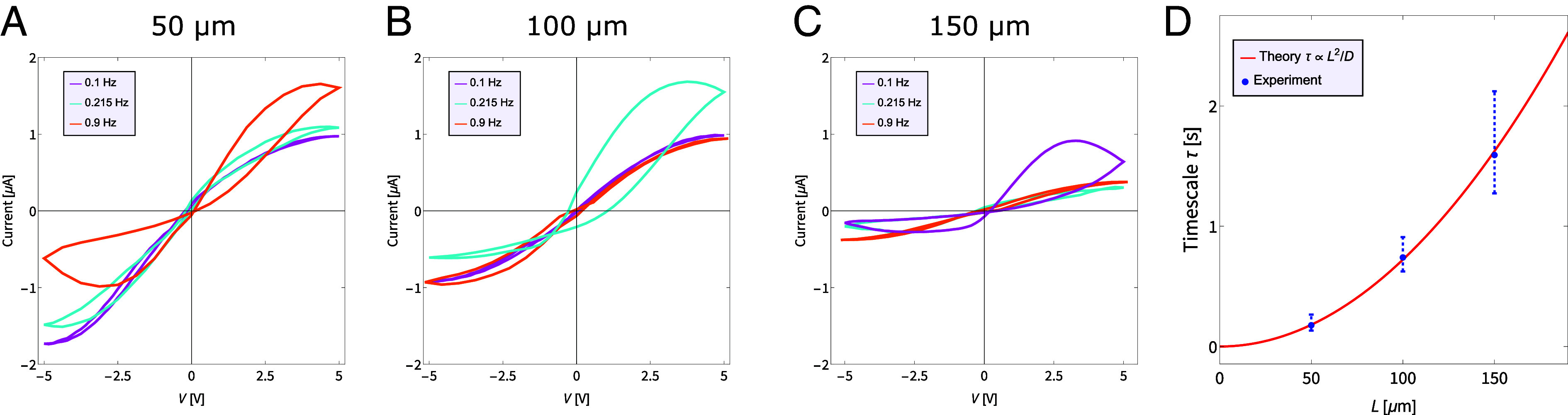
Voltage-driven concentration polarization occurs over a diffusionlike timescale. Experimental *I*–*V* curves for channels of length (*A*) 50μm, (*B*) 100μm, and (*C*) 150μm for a sinusoidal potential with amplitude 5 V and frequencies of 0.1 Hz (magenta), 0.215 Hz (cyan), and 0.9 Hz (orange). Measurements were also conducted for intermediate frequencies of 0.01, 0.05, 0.075, 0.1, 0.125, 0.175, 0.215, 0.255, 0.6, 0.9, 1.2, and 2 Hz, shown in *SI Appendix*. (*D*) Memory timescale τ for all channels determined by the frequency $f_{\;\text{max}}$ for which the enclosed area inside the loop is maximal via the relation $2\pi f_{\;\text{max}}\tau =1$ (51). The measured frequencies around each $f_{\;\text{max}}$ yield upper and lower bounds, shown here as error bars.

The typical timescale τ over which the conductance memory is retained is an important property of memristive systems and the ability to incorporate a wide range of timescales is desirable (27). As per Eq. **1**, we predict that the memory timescale of a device can freely be chosen from a wide range of options by constructing it with the appropriate length L or radii $R_{t}/R_{b}$, here we focus on the L-dependence. The prediction that τ is determined by a diffusionlike time $\propto L^{2}/D$, despite the channel being voltage driven, forms a specific, nontrivial, and easily falsifiable prediction. To test this we fabricated channels of lengths 50, 100, and 150μm and determined for all three at which frequency $f_{\;\text{max}}$ of an applied sinusoidal voltage the area enclosed in the hysteresis loop in the *I*–*V* diagram is maximal. Using the relation $2\pi f_{\;\text{max}}\tau =1$, we can find an estimate for the timescale τ (51), and check whether $\tau \propto L^{2}$. The natural relation $2\pi f_{\;\text{max}}\tau =1$ entails that maximal hysteresis is observed when the voltage changes over the typical memory time τ, i.e., providing enough time for the conductance to change, but not enough to reach its steady-state. We mathematically derived this relation for general memristors described by Eqs. **2** and **3** when a sinusoidal voltage is applied (51). Additionally, since the equilibrium channel conductance is inversely proportional to the channel length $g_{0}\propto 1/L$ we expect the overall current, and thus the overall loop area, to decrease for longer L at the same voltage. In our experiments, we indeed find that channels of different lengths respond to different frequencies, and show overall decreasing conductances for increasing L, as can be seen by the various hysteresis loops in Fig. 3*A*–*C*. By comparing the different values for $f_{\;\text{max}}$ we not only confirm that $f_{\;\text{max}}^{-1}\propto L^{2}$, but that quantitatively we have good agreement with the theory. Thus, by manufacturing channels of different lengths, facile via the flexible fabrication process, a wide range of memory timescales can be achieved. Therefore our device offers a versatility important for tasks that require processing of signals over various timescales (27).

## 2. Aqueous Reservoir Computing

The short-term memory properties of our fluidic memristor, with a memory retention time that can easily be chosen for each device from a wide range of options as shown in Fig. 3, make it a promising candidate for performing reservoir computing, a brain-inspired framework that leverages a fixed dynamic network, or “reservoir,” to transform complex temporal input data into an output that can be easily classified (31). Unlike traditional neural network approaches, where the full network needs to be trained, in reservoir computing only a comparatively simple read-out function (here a single-layer neural network) that classifies the output of the reservoir requires training (28). These properties have driven interest in reservoir computing for analyzing a variety of temporal signals (28, 30). Generally, a device needs two properties for it to be applicable in reservoir computing, i) a short-term memory and ii) nonlinear dynamics (30), which our device satisfies as shown in Fig. 1*F*.

**Fig. 4.**
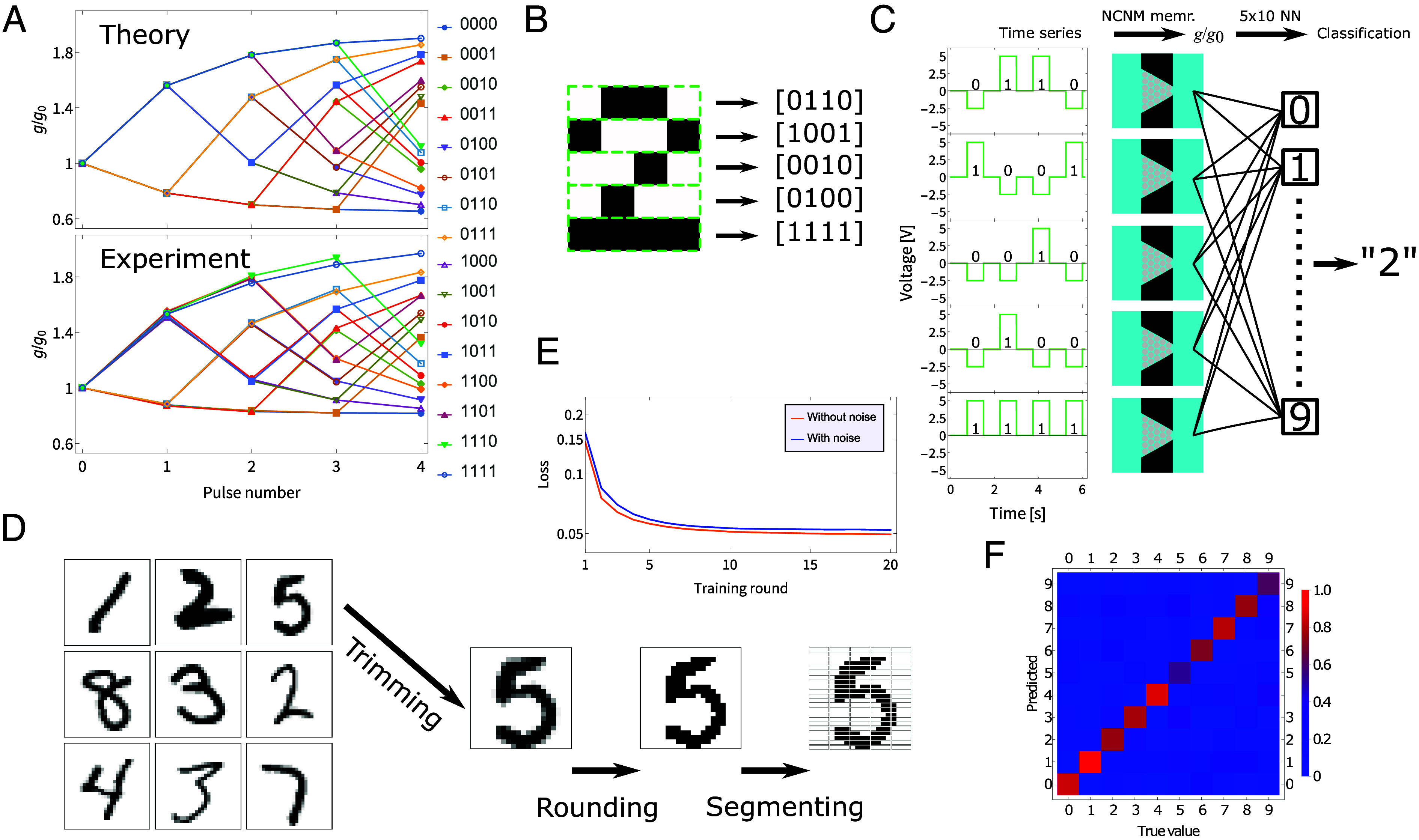
Our iontronic memristor as a reservoir computing element. (*A*) Theoretical prediction (*Top*) and experimental observation (*Bottom*) of the relative change in conductance as a response to the $2^{4}=16$ different possible bit-strings, where a “0” and “1” correspond to pulses of −2.5V and 5V, respectively. Three separate devices were used to find the average conductance and typical variation in response to each unique voltage train. (*B*) Depiction of how a “2” can be transformed into five distinct bit-strings (other digits are depicted in *SI Appendix*). (*C*) Schematic of how the number 2 from (*B*) is translated to 5 voltage trains, yielding 5 conductance values after the fourth (last) pulse. The conductances and variabilities from (*A*) were then used to train a single-layer fully connected 5×10 neural network in silico, that converts the conductances to a classification of the number 2. (*D*) Nine examples of handwritten numbers from the MNIST database (52), where the (originally 28×28 pixel) images are trimmed to 20×22 pixel images, the grayscales are rounded to either white or black pixels and then the image is segmented into 110 bit-strings. (*E*) The loss function (mean squared loss) during training per training round when the experimentally found noise, which is experimentally quantified using the (device-to-device) variabilities found in our result in (*A*), of the devices is not taken into account (orange) and when it is taken into account (blue). (*F*) The confusion matrix on a test set of 2,000 samples, showing an overall accuracy of 81%, comparable with recent reported results using more conventional platforms (32, 33).

To demonstrate the reservoir computing capabilities of our fluidic memristor, we carry out an established benchmarking protocol of classifying handwritten numbers using reservoir computing (32, 33). To build up to this, we first employ a standard method of separating 4-bit strings and show that our memristor already performs remarkably well at this initial task compared to previous results using more conventional platforms (34, 35). All $2^{4}=16$ combinations are translated into a series of voltage pulses with a duration of 0.75s, separated by intervals of 0.75s, where a 0 and a 1 correspond to a voltage of −2.5 V and 5 V, respectively. In theory, applying the voltage pulse trains should yield 16 distinct conductance time traces g(t) shown in Fig. 4*A*, *Top*, effectively mapping the 16 possible input patterns onto the 16 different conductance values after the fourth pulse, purely by virtue of the device properties. Excitingly, when we apply this protocol in experiments we find the predicted 16 distinct conductance signatures as shown in Fig. 4*A*, *Bottom*, featuring the exact same ordering of conductance values and quantitatively similar changes in conductances, once more highlighting the predictive power of the theory. To obtain Fig. 4*A*, *Bottom*, all 16 different voltage pulse trains were repeated twice on three different devices, producing six measurements in total and yielding the average conductances shown in Fig. 4*A*, *Bottom*. All six runs displayed essentially the same behavior, producing a spread of conductances after the fourth write-pulse for each pulse train, with SD of around $g/g_{0}∼$ 0.06 to 0.26 (complete list in *SI Appendix*). Individual device variability is lower, with typically SD of several $g/g_{0}∼$ 0.01 and no more than 0.16. As we perform an analog computing method, rather than discrete logic, the possible overlap between measured conductances is not an issue and the various time series can reliably encode (handwritten) numbers, as we show next.

To illustrate how the results shown in Fig. 4*A* can be leveraged to classify more complex data inputs with an explanatory example, let us consider the simple single-digit numbers 0 to 9, represented by black and white 4×5 pixel images. By converting a row of 4 pixels to a string of bits by letting a white pixel correspond to a 0 and a black pixel to a 1, we can encode the entire image with five strings of 4 bits, as shown in Fig. 4*B* for the number 2 (other digits are shown in *SI Appendix*). These bit-strings then generate five distinct signature outputs, as we saw in Fig. 4*A*. A single-layer fully connected 5×10 neural network is then trained in silico to classify the five measured conductances as numbers. This protocol is schematically illustrated in Fig. 4*C*. Other types of simple readout functions could possibly also suffice. We trained our read-out network in silico using the results shown in Fig. 4*A*, *Bottom*. To incorporate the (device-to-device) variability, each individual pulse was subject to some noise newly drawn from a normal distribution with mean 0 and SD given by the experimentally determined SD for that specific voltage train. During training, we repeated this process 100 times for each of the numbers 0 to 9, achieving perfect classification of all 10 digits with noise-free inference measurements. If we also take the noise into account during inference, we still achieve an overall accuracy of 95%, highlighting the system’s robustness against noise. Note that actual training is only performed on a simple and small neural network, that would otherwise not be capable of handling temporal inputs, while the “hard” work of separating the time-dependent signals is handled by the internal physics of our fluidic memristor. Ultimately, this successful classification of simple digit images serves as an explanatory proof-of-concept for the broader application of performing complex time-dependent data analysis tasks.

Building on our previous experiments with classifying simple digit images, we go a step further by classifying handwritten numbers from the well-known MNIST database. This database contains a large dataset of 28×28 pixel images of handwritten numbers and has become a standard dataset to test and demonstrate the classification capabilities of machine learning methods (52). We first converted each image into a 20×22 pixel black and white image by trimming the edges off and rounding the grayscales to either black or white pixels, as depicted in Fig. 4*D*. Each image was then sectioned into pixel rows of four pixels, which can be encoded with a voltage pulse train as outlined before, leading to a total of 110 conductance states per image. The readout function consists of a single-layer fully connected 110×10 neural network, trained on a dataset of 20,000 samples. The training incorporated the conductance response and device-to-device noise using the results shown in Fig. 4*A*, with the noise taken into account like we did for the classification 4×5 pixel digits. The noise hardly had any effect on overall accuracy, as can be seen by the nearly identical decrease of the loss function during training in Fig. 4*E*. Via this rudimentary straightforward protocol, we achieved an accuracy of 81% on a test set of 2,000 samples, comparable with earlier reported accuracies of 83% and 85.6% resulting from the same protocol using solid-state memristors (32, 33). In Fig. 4*F* the classification is schematically depicted in a confusion matrix, where we see how often each combination of true and predicted numbers occurred in the test set.

Our successful implementation of an aqueous iontronic device as a synaptic element for reservoir computing with a performance that is (at least) on par with more traditional platforms (32–35) is a promising demonstration of the potential that our fluidic platform offers for brain-inspired computing. The functionality extracted from our simple devices is remarkable, obviating the need for complicated circuits to distinguish the various time series of interest here, instead relying on the stable conductance modulation of individual devices. Consequently, as we advance toward circuits integrating multiple coupled NCNM devices, we anticipate that the robust individual device properties demonstrated here will enable the realization of expanded functionalities with relatively simple circuits.

## 3. Discussion and Conclusion

We implemented a fluidic iontronic volatile memristor as a synaptic element for neuromorphic reservoir computing, while the device relies on the same aqueous electrolyte medium and ionic signal carriers as biological neurons. Our memristor consists of a tapered microchannel that features a conducting network of nanochannels embedded in a rigid colloidal structure, forming a NCNM. Device fabrication is fast, cost-effective, and easy via an almost free-shaping soft-lithography process. The trait that underpins the conductance memory effect of the channel is its steady-state diode behavior, for which NCNM devices have shown excellent performance (36, 37), translating into a wide range of achievable conductances. Additionally, our device exhibited stable and reliable (dynamic) conductance modulation, enabling its use as a computing element. Moreover, the quadratic dependence of the memory timescale on the channel length offers a straightforward method to design a channel to feature a specific timescale, a sought-after feature for advancing neuromorphic computing capabilities (27).

Our memristor is inspired and supported by a comprehensive theory directly derived from the underlying physical equations of diffusive and electric continuum ion transport. We experimentally quantitatively verified the predictions of our theory on multiple occasions, among which the specific and surprising prediction that the memory retention time of the channel depends on the channel diffusion time, despite the channel being constantly voltage-driven. The theory exclusively relies on physical parameters, such as channel dimensions and ion concentrations, and enabled streamlined experimentation by pinpointing the relevant signal timescales, signal voltages, and suitable reservoir computing protocol. Additionally, we identify an inhomogeneous charge density as the key ingredient for iontronic channels to exhibit current rectification (provided they are well described by slab-averaged PNP equations). Consequently, our theory paves the way for targeted advancements in iontronic circuits and facilitates efficient exploration of their diverse applications.

For future prospects, a next step is the integration of multiple devices, where the flexible fabrication methods do offer a clear path toward circuits that couple multiple channels. Additionally, optimizing the device to exhibit strong conductance modulation for lower voltages would be of interest to bring electric potentials found in nature into the scope of possible inputs and reduce the energy consumption for conductance modulation. From a theoretical perspective, the understanding of the (origin of the) inhomogeneous space charge and the surface conductance is still somewhat limited. These contain (physical) parameters that are now partially chosen from a reasonable physical regime to yield good agreement, but do not directly follow from underlying physical equations. We also assume that the inhomogeneous ionic space charge distribution is constant, while it might well be voltage-dependent. Last, our theoretical model treats the complex porous structure in terms of slab-averages, thereby possibly missing out on detailed features. These constraints of the theoretical model could account for some of the discrepancies between theory and experiment, which is notable in the steady-state current in Fig. 1*B* and the decrease in conductance in Fig. 1*F*. For the purposes of this work, our current approach is sufficient, however, a more in-depth study could offer a more profound understanding of the interesting features of the channel.

In conclusion, in order to narrow the gap between the promise of aqueous iontronic neuromorphic computation and its implementation, our work demonstrates the capabilities of a fluidic memristor by employing it as an artificial synapse for carrying out neuromorphic reservoir computing. Temporal signals, in the form of voltage pulse trains, that together represent (handwritten) numbers were distinguished by individual channels for subsequent in silico classification with a simple readout function, demonstrating (at least) comparable performance to more conventional solid-state platforms (32–35). Additionally, the device is fabricated with a cost-effective easy soft-lithography process. The achieved computing properties are inspired and supported by a quantitative predictive theoretical model of the device dynamics. Consequently, our work establishes a solid foundation, both theoretically and experimentally, for future investigations into fluidic memristive systems and their application in aqueous neuromorphic computing architectures, paving the way for computing systems that more closely resemble the brain’s fascinating aqueous processes.

## Materials and Methods

The fabrication of microchannel and formation of the NCNM for the fluidic memristor is similar to previously reported methods (36, 37) and is described in *SI Appendix* in detail. A master for multilayered channels (target heights are 5 μm for shallow channel and 100 μm for deep) was created using a multistep UV exposure with negative photoresist (PR, SU-8 2005, 3050, Microchem Co.). After surface treatment of the master with (3,3,3-trifluoropropyl)silane (452807, Sigma-Aldrich) for easy separation, Polydimethylsiloxane (PDMS, Sylgard, Dow Corning Korea Ltd., Korea) was poured and cured by heating. The detached PDMS device was bonded with a slide glass. The formation of NCNM was formed by a self-assembly of homogeneous nanoparticles with negative surface charge in the desired shallow channel using Laplace pressure to halt the solvent at the base and evaporation of solvent. A close-packed fcc was formed by the growth of the ordered lattice induced by the evaporation.

## Supplementary Material

Appendix 01 (PDF)

## Data Availability

All study data are included in the article and/or *SI Appendix*.

## References

[1] A. Mehonic, A. J. Kenyon, Brain-inspired computing needs a master plan. Nature **604**, 255–260 (2022). 35418630 10.1038/s41586-021-04362-w

[2] C. D. Schuman , A survey of neuromorphic computing and neural networks in hardware. arXiv [Preprint] (2017). 10.48550/arXiv.1705.06963 (Accessed 19 September 2023).

[3] V. K. Sangwan, M. C. Hersam, Neuromorphic nanoelectronic materials. Nat. Nanotechnol. **15**, 517–528 (2020). 32123381 10.1038/s41565-020-0647-z

[4] C. D. Schuman , Opportunities for neuromorphic computing algorithms and applications. Nat. Comput. Sci. **2**, 10–19 (2022). 38177712 10.1038/s43588-021-00184-y

[5] D. B. Strukov, G. S. Snider, D. R. Stewart, R. S. Williams, The missing memristor found. Nature **453**, 80–83 (2008). 18451858 10.1038/nature06932

[6] L. Chua, Memristor, Hodgkin–Huxley, and edge of chaos. Nanotechnology **24**, 383001 (2013). 23999613 10.1088/0957-4484/24/38/383001

[7] J. Zhu, T. Zhang, Y. Yang, R. Huang, A comprehensive review on emerging artificial neuromorphic devices. Appl. Phys. Rev. **7**, 011312 (2020).

[8] L. Squire , Fundamental Neuroscience (Academic Press, ed. 3, 2008).

[9] A. Noy, S. B. Darling, Nanofluidic computing makes a splash. Science **379**, 143–144 (2023). 36634195 10.1126/science.adf6400

[10] S. H. Han, M. A. Oh, T. D. Chung, Iontronics: Aqueous ion-based engineering for bioinspired functionalities and applications. Chem. Phys. Rev. **3**, 031302 (2022).

[11] M. R. Powell, L. Cleary, M. Davenport, K. J. Shea, Z. S. Siwy, Electric-field-induced wetting and dewetting in single hydrophobic nanopores. Nat. Nanotechnol. **6**, 798–802 (2011). 22036811 10.1038/nnano.2011.189

[12] D. Wang , Transmembrane potential across single conical nanopores and resulting memristive and memcapacitive ion transport. J. Am. Chem. Soc. **134**, 3651–3654 (2012). 22313339 10.1021/ja211142e

[13] G. Paulo , Hydrophobically gated memristive nanopores for neuromorphic applications. Nat. Commun. **14**, 8390 (2023). 38110352 10.1038/s41467-023-44019-y PMC10728163

[14] P. Ramirez , Neuromorphic responses of nanofluidic memristors in symmetric and asymmetric ionic solutions. J. Chem. Phys. **160**, 044701 (2024). 38258920 10.1063/5.0188940

[15] S. H. Han, S. I. Kim, M. A. Oh, T. D. Chung, Iontronic analog of synaptic plasticity: Hydrogel-based ionic diode with chemical precipitation and dissolution. Proc. Natl. Acad. Sci. U.S.A. **120**, e2211442120 (2023). 36574693 10.1073/pnas.2211442120 PMC9910479

[16] P. Ramirez, V. Gómez, J. Cervera, S. Mafe, J. Bisquert, Synaptical tunability of multipore nanofluidic memristors. J. Phys. Chem. Lett. **14**, 10930–10934 (2023). 38033300 10.1021/acs.jpclett.3c02796

[17] T. Xiong , Neuromorphic functions with a polyelectrolyte-confined fluidic memristor. Science **379**, 156–161 (2023). 36634194 10.1126/science.adc9150

[18] P. Robin , Long-term memory and synapse-like dynamics in two-dimensional nanofluidic channels. Science **379**, 161–167 (2023). 36634187 10.1126/science.adc9931

[19] P. Robin, N. Kavokine, L. Bocquet, Modeling of emergent memory and voltage spiking in ionic transport through Angstrom-scale slits. Science **373**, 687–691 (2021). 34353953 10.1126/science.abf7923

[20] T. M. Kamsma, W. Q. Boon, T. ter Rele, C. Spitoni, R. van Roij, Iontronic neuromorphic signaling with conical microfluidic memristors. Phys. Rev. Lett. **130**, 268401 (2023). 37450821 10.1103/PhysRevLett.130.268401

[21] T. Kamsma, E. Rossing, C. Spitoni, R. van Roij, Advanced iontronic spiking modes with multiscale diffusive dynamics in a fluidic circuit. arXiv [Preprint] (2024). 10.48550/arXiv.2401.14921 (Accessed 26 January 2024).

[22] T. Emmerich , Nanofluidic logic with mechano-ionic memristive switches. *Nat. Electron.* 1–8 (2024), 10.1038/s41928-024-01137-9. PMC11045460 38681725

[23] B. Sabbagh, N. E. Fraiman, A. Fish, G. Yossifon, Designing with iontronic logic gates-from a single polyelectrolyte diode to an integrated ionic circuit. ACS Appl. Mater. Interfaces **15**, 23361–23370 (2023). 37068481 10.1021/acsami.3c00062 PMC10197067

[24] J. Li, M. Li, K. Zhang, L. Hu, D. Li, High-performance integrated iontronic circuits based on single nano/microchannels. Small **19**, 2208079 (2023). 10.1002/smll.202208079 36869414

[25] B. Xie , Perspective on nanofluidic memristors: From mechanism to application. Chem. Asian J. **17**, e202200682 (2022). 35994236 10.1002/asia.202200682

[26] A. Noy, Z. Li, S. B. Darling, Fluid learning: Mimicking brain computing with neuromorphic nanofluidic devices. Nano Today **53**, 102043 (2023).

[27] E. Chicca, G. Indiveri, A recipe for creating ideal hybrid memristive-CMOS neuromorphic processing systems. Appl. Phys. Lett. **116**, 120501 (2020).

[28] G. Tanaka , Recent advances in physical reservoir computing: A review. Neural Netw. **115**, 100–123 (2019). 30981085 10.1016/j.neunet.2019.03.005

[29] M. Cucchi , Reservoir computing with biocompatible organic electrochemical networks for brain-inspired biosignal classification. Sci. Adv. **7**, eabh0693 (2021). 34407948 10.1126/sciadv.abh0693 PMC8373129

[30] J. Cao , Emerging dynamic memristors for neuromorphic reservoir computing. Nanoscale **14**, 289–298 (2022). 34932057 10.1039/d1nr06680c

[31] M. Cucchi, S. Abreu, G. Ciccone, D. Brunner, H. Kleemann, Hands-on reservoir computing: A tutorial for practical implementation. Neuromorphic Comput. Eng. **2**, 032002 (2022).

[32] R. Midya , Reservoir computing using diffusive memristors. Adv. Intell. Syst. **1**, 1900084 (2019).

[33] C. Du , Reservoir computing using dynamic memristors for temporal information processing. Nat. Commun. **8**, 1–10 (2017). 29259188 10.1038/s41467-017-02337-y PMC5736649

[34] J. Pyo, S. Kim, Non-volatile and volatile switching behaviors determined by first reset in Ag/TaO_*x*_/TiN device for neuromorphic system. J. Alloy. Compd. **896**, 163075 (2022).

[35] D. Kim, J. Shin, S. Kim, Implementation of reservoir computing using volatile WO_*x*_-based memristor. Appl. Surf. Sci., 153876 (2022).

[36] E. Choi, C. Wang, G. T. Chang, J. Park, High current ionic diode using homogeneously charged asymmetric nanochannel network membrane. Nano Lett. **16**, 2189–2197 (2016). 26990504 10.1021/acs.nanolett.5b04246

[37] J. Kim, J. Jeon, C. Wang, G. T. Chang, J. Park, Asymmetric nanochannel network-based bipolar ionic diode for enhanced heavy metal ion detection. ACS Nano **16**, 8253–8263 (2022). 35442631 10.1021/acsnano.2c02016

[38] A. Mani, T. A. Zangle, J. G. Santiago, On the propagation of concentration polarization from microchannel–nanochannel interfaces. Part I: Analytical model and characteristic analysis. Langmuir **25**, 3898–3908 (2009). 19275187 10.1021/la803317p PMC4816500

[39] A. Mani, M. Z. Bazant, Deionization shocks in microstructures. Phys. Rev. E **84**, 061504 (2011). 10.1103/PhysRevE.84.061504 22304094

[40] W. Q. Boon, T. E. Veenstra, M. Dijkstra, R. van Roij, Pressure-sensitive ion conduction in a conical channel: Optimal pressure and geometry. Phys. Fluids **34**, 101701 (2022).

[41] T. M. Kamsma, W. Q. Boon, C. Spitoni, R. van Roij, Unveiling the capabilities of bipolar conical channels in neuromorphic iontronics. Faraday Discuss. **246**, 125–140 (2023). 37404026 10.1039/d3fd00022b PMC10568261

[42] M. Schmuck, M. Z. Bazant, Homogenization of the Poisson–Nernst–Planck equations for ion transport in charged porous media. SIAM J. Appl. Math. **75**, 1369–1401 (2015).

[43] T. A. Zangle, A. Mani, J. G. Santiago, On the propagation of concentration polarization from microchannel–nanochannel interfaces. Part II: Numerical and experimental study. Langmuir **25**, 3909–3916 (2009). 19275188 10.1021/la803318e PMC4816496

[44] L. Jubin, A. Poggioli, A. Siria, L. Bocquet, Dramatic pressure-sensitive ion conduction in conical nanopores. Proc. Natl. Acad. Sci. U.S.A. **115**, 4063–4068 (2018). 29610303 10.1073/pnas.1721987115 PMC5910861

[45] V. S. Markin, A. G. Volkov, L. Chua, An analytical model of memristors in plants. Plant Signal. Behav. **9**, e972887 (2014). 25482769 10.4161/15592316.2014.972887 PMC4622502

[46] L. Chua, If it’s pinched it’s a memristor. Semicond. Sci. Technol. **29**, 104001 (2014).

[47] W. Brown, M. Kvetny, R. Yang, G. Wang, Selective ion enrichment and charge storage through transport hysteresis in conical nanopipettes. J. Phys. Chem. C **126**, 10872–10879 (2022).

[48] Z. Rotman, P. Y. Deng, V. A. Klyachko, Short-term plasticity optimizes synaptic information transmission. J. Neurosci. **31**, 14800–14809 (2011). 21994397 10.1523/JNEUROSCI.3231-11.2011 PMC6703406

[49] L. Abbott, W. G. Regehr, Synaptic computation. Nature **431**, 796–803 (2004). 15483601 10.1038/nature03010

[50] P. Y. Deng, V. A. Klyachko, The diverse functions of short-term plasticity components in synaptic computations. Commun. Integr. Biol. **4**, 543–548 (2011). 22046457 10.4161/cib.4.5.15870 PMC3204123

[51] T. M. Kamsma, R. van Roij, C. Spitoni, A simple mathematical theory for Simple Volatile Memristors and their spiking circuits. ResearchGate [Preprint]. 10.13140/RG.2.2.13242.40640. Deposited 26 February 2024.

[52] L. Deng, The MNIST database of handwritten digit images for machine learning research [best of the web]. IEEE Signal Process. Mag. **29**, 141–142 (2012).

