# Supplementary material for: Brain-inspired computing with fluidic iontronic nanochannels
Source: Proc Natl Acad Sci U S A. 2024 Apr 24;121(18):e2320242121. doi: 10.1073/pnas.2320242121 (PMC11067030; doi:10.1073/pnas.2320242121)
Supplement: Supplementary file 1 — Appendix 01 (PDF) [file pnas.2320242121.sapp.pdf]

Supplemental Information for: Brain-inspired computing with fluidic iontronic nanochannels

T. M. Kamsma,[1,2,*,†] J. Kim,[3,*] K. Kim,[3] W. Q. Boon,[1] C. Spitoni,[2] J. Park,[3,†] and R. van Roij[1,†]

[1]*Institute for Theoretical Physics, Utrecht University, Princetonplein 5, 3584 CC Utrecht, The Netherlands*
[2]*Mathematical Institute, Utrecht University, Budapestlaan 6, 3584 CD Utrecht, The Netherlands*
[3]*Department of Mechanical Engineering, Sogang University, 35 Baekbeom-ro (Sinsu-dong), Mapo-gu, Seoul 04107, Republic of Korea*

(Dated: March 28, 2024)

1 Theory

1.1 Poisson-Nernst-Planck equations

We consider ionic transport through a triangular tapered channel of uniform height H, length L, and widths $2R_\mathrm{t}$ and $2R_\mathrm{b}$ at the (smaller) tip and the (larger) base, i.e. $R_\mathrm{t} < R_\mathrm{b}$, as illustrated in Fig. 1(a) of the main text. In our experiments we have $L = 150\ \mu$m (unless stated otherwise), $R_\mathrm{t} = 5\ \mu$m and $R_\mathrm{b} = 100\ \mu$m. We introduce the axial coordinate x, with $x = 0$ at the base and $x = L$ at the tip, and the cartesian width-coordinate $y \in [0, R(x)]$, with $y = 0$ the symmetry axis and $y = R(x)$ the half width of the channel given by $R(x) = R_\mathrm{b} - (R_\mathrm{b} - R_\mathrm{t})x/L$ for $x \in [0, L]$. In the channel the height coordinate z lies in the interval $z \in [-H/2, H/2]$. The channel is filled with a rigid close-packed fcc crystal of charged colloidal spheres (in the experiments with packing fraction $\eta \simeq 0.74$, radius $a = 100$ nm, and zeta potential $\psi_0 \approx -39$ mV). The channel connects two large and deep aqueous reservoirs containing a 1:1 electrolyte at room temperature with salt bulk concentration ρ_b (in the experiments $\rho_\mathrm{b} = 10$ mM KCl with a Debye length $\lambda_\mathrm{D} = 3.1$ nm). The ionic transport takes place through the electrolyte that fills the space between the colloids in the colloidal crystal and is driven by a time-dependent voltage $V(t)$. We define the applied voltage $V = V(L) - V(0)$ to be the voltage at the tip minus the voltage at the base, such that conductance enhancement always occurs when $V > 0$, regardless of which side is grounded. In the main text we have a grounded tip so we apply $V_\mathrm{app}(t) = -V(t)$ at the base. The transport is described in terms of the Poisson-Nernst-Planck (PNP) equations, that relate the electrostatic potential $\Psi(x,y,z,t)$ and the cationic and anionic concentrations $\rho_+(x,y,z,t)$ and $\rho_-(x,y,z,t)$, respectively, to the ionic fluxes $\mathbf{j}_\pm(x,y,z,t)$. In the space between the colloidal spheres we write

$$\nabla^2\Psi = -\frac{e}{\varepsilon}(\rho_+ - \rho_-), \tag{S1}$$

$$\frac{\partial \rho_\pm}{\partial t} + \nabla \cdot \mathbf{j}_\pm = 0, \tag{S2}$$

$$\mathbf{j}_\pm = -D\left(\nabla\rho_\pm \pm \rho_\pm \frac{e\nabla\Psi}{k_\mathrm{B}T}\right), \tag{S3}$$

where e denotes the elementary charge, $\varepsilon = 80.23\varepsilon_0$ the dielectric constant of water at room temperature T, k_B the Boltzmann constant, and D the ionic diffusion coefficient that we take equal for the cations and the anions. The electrostatics is accounted for by the Poisson equation (S1), the conservation of ions by the continuity equation (S2), and the combination of Fickian diffusion and Ohmic conduction by the Nernst-Planck equation (S3). We neglect electro-osmotic fluid flow, which we expect to be relatively small due to the narrow constrictions of the geometry. The system of equations (S1)-(S3) is closed upon imposing blocking boundary conditions on all solid walls, $\mathbf{n} \cdot \mathbf{j}_\pm = 0$, with $\mathbf{n}$ the (inward) normal on the walls of the channel and the colloids, together with Gauss' law $\mathbf{n} \cdot \nabla\Psi = -e\sigma/\varepsilon$ with σ the surface charge density (on the wall and on the colloidal surfaces). We also impose that $\rho_\pm(x,y,z,t)$ equals the bulk concentration ρ_b at the far end of either reservoir, that $\Psi(0,y,z,t) = 0$ and $\Psi(L,y,z,t) = V(t)$ to account for the applied potentials.

The resulting closed set of PNP equations and boundary conditions can in principle be solved numerically by finite-element methods. The geometry of a colloidal crystal in a tapered channel, however, is computationally challenging as it requires a spatial resolution on the nm length scale of the electric double layer, on the 1-100 nm length scale of the pore structure in between the

* These two authors contributed equally to this work

† Corresponding author

colloidal particles, and on the 10-100 μm length scale of the channel dimensions. By treating the complex porous structure homogeneous medium, these equations were modified and successfully solved numerically to describe the simplified physics inside the type of channel of interest here [1], however at a considerable computational cost and no analytic insights. Instead of a computationally costly numerical approach, we will derive analytical results straight from the Nernst-Planck equation (S3) to obtain an analytic approximation for the channel dynamics. This will yield a computationally significantly cheaper theoretical model which can be treated analytically to investigate the origin of the ion current rectifying properties of the channel, and to predict features such as its conductance memory properties.

1.2 Slab-averaged electric field, space charge, and salt concentration

Our theoretical approach is based upon a methodology that we successfully developed and applied recently to quantitatively explain the steady-state and dynamic conductance properties of simpler channels filled with a homogeneous aqueous electrolyte [2–4]. Here we show that this methodology can be extended to the nanoporous channel network that characterizes the channel we study in this work. The colloidal structure within the channel forms a (nearly) close-packed face centered cubic (fcc) crystal at a volume fraction $\eta \simeq 0.74$ as we saw before. With a colloid radius $a = 100$ nm, this means that the pores through which ions can be transported have diameters as large as several tens of nm in the octahedral and tetrahedral holes of the fcc-lattice [5, 6], which is much larger than the Debye length of $\lambda_{\mathrm{D}} \approx 3.1$ nm that characterises the thickness of the electric double layers. In other words, the channels are mostly in the regime of non-overlapping and hence fully developed thin electric double layers, bringing us into the scope of area-averaging techniques [2, 4, 7, 8]. Specifically, this justifies the same underlying assumption as in Refs. [2–4] that the local and voltage-dependent total salt concentration $\rho_{\mathrm{s}} \equiv \rho_+ + \rho_-$, the total ionic space charge density $\rho_e = \rho_+ - \rho_-$, and the local electric potential Ψ can faithfully be represented by the $y-z$-slab-averaged functions $\overline{\rho}_{\mathrm{s}}(x,V)$, $\overline{\rho_e}(x)$, and $\overline{\Psi}(x)$, respectively, where we recall that the lateral coordinate $x \in [0, L]$ runs from base to tip. Here we explicitly denote the dependence of $\overline{\rho}_{\mathrm{s}}$ on V, while refraining from denoting the explicit (linear) dependence of $\overline{\Psi}$ on V below for notational convenience. While we expect also a V-dependence of $\overline{\rho_e}$ in a full calculation, we will restrict ourselves to a V-independent form below.

If the slab-averaged electric field lines cannot escape the tapered channel, a realistic assumption in our experiments as the dielectric constant of water is much higher than that of the wall-material, then the slab-averaged electric field component $-\partial_x\overline{\Psi}(x)$ must be proportional to $1/R(x)$ on the basis of charge neutrality on the length scale beyond the Debye length. Since we define $V = V(L) - V(0)$, i.e. the tip minus the base voltage, the applied voltage also satisfies $\int_0^L \partial_x\overline{\Psi}(x)\mathrm{d}x = V$. Combining the scaling with this property, we find with $\Delta R = R_{\mathrm{b}} - R_{\mathrm{t}}$ that

$$\partial_x\overline{\Psi}(x) = \frac{\Delta R V}{L\ln\left(\frac{R_{\mathrm{b}}}{R_{\mathrm{t}}}\right)R(x)}. \tag{S4}$$

This slab-averaged electric field in the channel is therefore proportional to the applied field V/L and gets progressively stronger closer to the tip. The total steady-state salt flux $\mathbf{j}_{\mathrm{s}}(x,y,z) = \mathbf{j}_+(x,y,z,t) + \mathbf{j}_-(x,y,z)$ can now be integrated over slabs in the y and z direction to obtain for the x-component of the total salt flux $J_x(x) = \int_{-R(x)}^{R(x)}\int_{-H/2}^{H/2}\mathbf{j}_{\mathrm{s}}(x,y,z)\cdot\hat{\mathbf{x}}\mathrm{d}y\mathrm{d}z$ through the channel

$$J_x(x) = -D\varepsilon_{\mathrm{fcc}}\left(2R(x)H\partial_x\overline{\rho}_{\mathrm{s}}(x,V) + 2R(x)H\overline{\rho_e}(x)\frac{e\partial_x\overline{\Psi}(x)}{k_{\mathrm{B}}T}\right). \tag{S5}$$

Here we introduced the porosity $\varepsilon_{\mathrm{fcc}} = 1-\eta$ to take the volume into account that is excluded to the electrolyte by the colloidal fcc crystal, where we assume the colloidal particles to be impenetrable to the electrolyte. As we take slab averages one would expect an area term, instead of the porosity. However, microscopically, the available electrolyte area through which the ions can diffuse in the slab has a periodicity in x that is dictated by the lattice spacing, which is much smaller than the channel length L and can hence be ignored in our slab-averaged description. Therefore we consider each slab to have the same available surface area for ions, which in this simplified one-dimensional view is the porosity $\varepsilon_{\mathrm{fcc}}$ [9].

Eq. (S5) depends on the slab-averaged ionic space charge density $\overline{\rho_{\mathrm{e}}}(x)$, that picks up contributions from the electric double layers (EDLs) around the charged colloidal spheres. Given that EDLs have spatial extensions as small as the Debye length (here $\lambda_{\mathrm{D}} = 3.1$ nm) around the colloidal spheres (here of radius $a = 100$ nm), one might expect the slab-averaged space charge density to be a constant on the much larger length scale of the channel, with a magnitude $\propto \eta Z$ with Z the charge of a colloid. Interestingly, however, earlier measurements presented in Ref. [1] on channels nearly identical to the ones we study here convincingly showed a heterogeneous rather than a homogeneous space charge density that could well be fitted by the monotonic functional form

$$\overline{\rho_{\rm e}}(x) = \frac{\rho_{\rm e,t}}{1-(1-\rho_{\rm e,t}/\rho_{\rm e,b})(L-x)/L}, \tag{S6}$$

where $\rho_{\rm e,t}$ and $\rho_{\rm e,b}$ are the space charge density at the tip and the base, respectively, which may differ from each other even at zero applied voltage [1]. In this work we have $\rho_{\rm e,t} = 8.85$ mM and $\rho_{\rm e,b} = 8.00$ mM for the steady-state results of Fig. 1(b) and $\rho_{\rm e,b} = 6.3$ mM for the other (dynamic) results, the choice for these values is further discussed in Sec. 2. Whereas we take the functional form of Eq.(S6) as experimental input from now on, the reason for this ionic charge heterogeneity on length scales of the channel length remains an interesting open question. Our hypothesis, that we underpin with standard Poisson-Boltzmann calculations of a single colloid in a spherical Wigner-Seitz cell in Sec. 1 .5, shows that a reduction of the colloidal packing fraction from 0.74 to 0.73 can already increase the colloidal charge density (and hence the average ionic charge in the EDLs) by as much as $\sim 20\%$ at a fixed zeta potential. Thus, a small heterogeneity of the colloidal packing fraction from tip to base could provide a microscopic explanation for the spatial dependence of $\overline{\rho_{\rm e}}(x)$ by assuming macroscopic electroneutrality [7, 9]. Given the device fabrication (see Sec. 3) such a small asymmetry of η between tip and base is quite well possible although such a small deviation in packing fraction is difficult to observe experimentally.

With Eqs. (S4) and (S6) every component of Eq. (S5) is known except for $\overline{\rho}_{\rm s}(x,V)$. We can use Eq. (S5) for $J_x(x)$ to find the steady-state salt concentration $\overline{\rho_{\rm s}}(x,V)$ explicitly by imposing the steady-state condition $\partial_x J_x(x) = 0$. This yields a differential equation for $\overline{\rho_{\rm s}}(x,V)$, that can be solved analytically after inserting Eqs.(S4) and (S7), yielding

$$\overline{\rho_{\rm s}}(x,V) = 2\rho_{\rm b} - \rho_{\rm e,b}\frac{eV}{k_{\rm B}T}\frac{\Delta R}{R_{\rm b}}\frac{\ln\left(\frac{R_{\rm b}}{R_{\rm t}}\right)\ln\left(\frac{\rho_{\rm e,t}}{\rho_{\rm e,b}}\frac{L-x}{L}+x/L\right)+\ln\left(\frac{R(x)}{R_{\rm t}}\right)\ln\left(\frac{\rho_{\rm e,b}}{\rho_{\rm e,t}}\right)}{\ln^2\left(\frac{R_{\rm b}}{R_{\rm t}}\right)(\rho_{\rm e,b}/\rho_{\rm e,t}-R_{\rm t}/R_{\rm b})}. \tag{S7}$$

We note that H, D, and $\varepsilon_{\rm fcc}$ do not appear in Eq. (S7) since these drop out of the underlying differential equation $\partial_x J_x(x) = 0$. More importantly, $\overline{\rho_{\rm s}}(x,V)$ is voltage-dependent, resulting in the voltage-dependent channel conductance we derive next.

1.3 Static channel conductance

The tapered microchannels of our interest are well known to exhibit ionic rectification properties characterised by a static conductance $g_\infty \equiv I(V)/V$ that is a nontrivial function of the applied static voltage V. By viewing the slabs of thickness dx at $x \in [0,L]$ as a series of resistors with resistivities $\propto {\rm d}x/\rho_{\rm s}(x)$, one can write the static conductance of the channels of present interest as

$$g_\infty(V) = g_0\int_0^L \overline{\rho}_{\rm s}(x,V){\rm d}x/(2\rho_{\rm b}L), \tag{S8}$$

where we made an approximation compared to the more accurate dependence on $L/\int_0^L(\overline{\rho}_{\rm s}(x,V))^{-1}{\rm d}x$, which reduces computational complexity and yields mostly the same results [2–4]. The V-dependence stems from the salt concentration dependence on V as given by Eq.(S7). As in Ref. [4], we replace $\overline{\rho}_{\rm s}(x,V)$ by $\max[0.2\rho_{\rm b},\overline{\rho}_{\rm s}(x,V)]$ in the actual (numerical) evaluations of Eq. (S8) in order to account for the possibility of unphysical negative concentrations that could follow from Eq.(S7) at strongly negative voltages in part of the density profiles. (In this regime the underlying assumption that the Debye length is much larger than than the channel dimensions breaks down).

The reference (zero-voltage) conductance g_0 of the channel can, in direct analogy with recent results from Refs. [2–4], be written as

$$g_0 = 2\rho_{\rm b}\varepsilon_{\rm fcc}eD\frac{2\Delta RH}{L\ln(\frac{R_{\rm b}}{R_{\rm t}})}\frac{e}{k_{\rm B}T}+g_{\rm s} = 2\rho_{\rm b}\frac{e^2D}{k_{\rm B}T}\varepsilon_{\rm fcc}\frac{2\Delta RH}{L\ln(\frac{R_{\rm b}}{R_{\rm t}})}\left[1+\frac{4\lambda_{\rm D}}{R_{\rm pore}}\left(\cosh\left(\frac{e\psi_0}{2k_{\rm B}T}\right)-1\right)\right], \tag{S9}$$

which includes the volumetric contribution $\propto \varepsilon_{\rm fcc}$ that depends on the channel-geometry parameters calculated with the total charge flux $\int_{-R(x)}^{R(x)}\int_{-H/2}^{H/2}[\mathbf{j}_+(x,y,z)-\mathbf{j}_-(x,y,z)]\cdot\hat{\mathbf{x}}{\rm d}y{\rm d}z$. Additionally we also consider a surface contribution $\propto \lambda_{\rm D}/R_{\rm pore}$, with $R_{\rm pore}$ the effective radius of the pores embedded in the fcc crystal, which accounts for the excess conductivity due to the excess salt concentration in the colloidal EDLs [10] and which we determine below. This surface term is of direct relevance here due to the large internal surface in the channel as a result of the colloidal structure. However, the internal structure of the fcc crystal is complex with pores of varying sizes and shapes, and regions with fully developed EDLs in the pores with the size of several tens

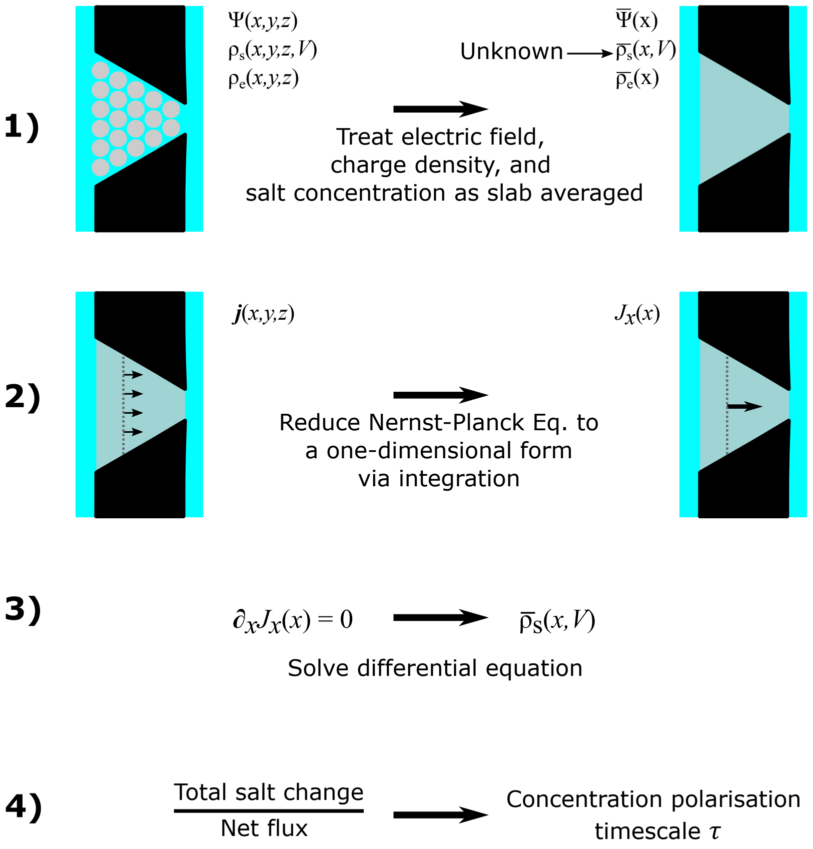

Fig. S1. Schematic depiction of the steps taken in our theoretical analysis, involving 1) the slab-averaging assumption, 2) the integration of the salt flux over each slab to reduce the full three-dimensional Nernst-Planck equation to its one-dimensional slab-averaged form as in Eq. (S5), and 3) the solution of the steady-state condition of a divergence-free flux to obtain Eq. (S7). Lastly, 4) the combination of the salt concentration and the salt flux to extract a timescale for the salt concentration polarisation as detailed in Sec. 1 .4.

of λ_D neighboured by regions where the EDLs are not fully developed since the shortest distance between colloids is smaller than a few λ_D. Therefore, the surface conductance of the channel is a highly non-trivial property and investigating this in full detail falls outside the scope of this work. Instead we will employ an effective method where we treat the internal pores as a channel of radius R_{pore}, with a total slab surface area $2\varepsilon_{\text{fcc}}R(x)H$. This approach then yields Eq. (S9) [10]. We find good agreement with the experiments if we take $R_{\text{pore}} = 2\lambda_D = 0.06a$. Although this seems somewhat small compared to the radii of up to $\sim 10\lambda_D$ for the larger pores, it is still in a reasonable regime given the fact that many regions in a close-packed fcc crystal feature much smaller distances between colloids. For the parameters used in the experiment (see Sec. 2) we have that $g_s/g_0 \approx 0.38$, so the conductance is still mostly dictated by bulk conductance, as expected with the relatively large pores compared to the Debye length.

The combination of Eq. (S8) and Eq. (S9) provides us with the steady-state conductance function $g_\infty(V)$ used in the main text.

1.3.1 Space charge density inhomogeneity is a salt source or sink term

The methodology we employ in Sec. 1 .2 to derive salt concentration polarisation can provide insights into designing new devices that also exhibit salt accumulation and depletion. As shown in Sec. 1 .3, the presence of salt concentration polarisation affects the channel conductance and will hence lead to current rectification. Our theoretical model explains current rectification in the colloid-filled channels we present here, but other types of channels also exhibit current rectification as a result of an inhomogeneous space charge [11, 12]. Our theoretical description might provide insights into such other systems as well, provided some assumptions are satisfied. (i) The underlying PNPS equations only describe continuum transport, so sub-nm or atomic scale systems [13, 14] will fall outside the scope of this theoretical framework. (ii) The radial dependence of the potential and the concentrations must be relatively weak and/or short ranged, such that the slab-averaged salt-concentration $\overline{\rho}_s(x,V)$ and electric field $-\partial_x\overline{\Psi}(x)$ are fair approximations. For instance, a single channel with strongly overlapping electric double layers

throughout the entire channel will exhibit a significant salt concentration profile in the radial direction of the channel, possibly resulting in physical phenomena that a slab average $\overline{\rho}_{\rm s}(x,V)$ does not take into account.

Eq. [S5] can be generalised to

$$J_x(x) = -Da\left(A(x)\partial_x\overline{\rho}_{\rm s}(x,V) + Q_e(x)\frac{e\partial_x\overline{\Psi}(x)}{k_{\rm B}T}\right) = -Da\left(A(x)\partial_x\overline{\rho}_{\rm s}(x,V) + A(x)\overline{\rho_e}(x)\frac{e\partial_x\overline{\Psi}(x)}{k_{\rm B}T}\right), \tag{S10}$$

with $Q_e(x)\mathrm{d}x$ the total ionic charge in a slab of infinitesimal thickness dx with volume $A(x)\mathrm{d}x$ at location x, and where we define $\overline{\rho_e}(x) \equiv Q_e(x)/A(x)$ the slab-averaged ionic charge density. Eq. (S10) describes a channel with cross-sectional area $A(x)$ and the average area per slab available for ions a (which would be 1 for materials without any solid obstructions for the ions), where we remark that in the simplified one-dimensional view of treating each slab to feature the same available area one can use the porosity as we did in Eq. (S5) [9]. In the present channel geometry we have $A(x) = 2HR(x)$, but Eq. (S10) could also apply to hourglass shaped channels [12], T-shaped channels [11], or conical channels [2, 4]. If macroscopic charge neutrality is ensured within the channel, e.g. in the case of thin electric double layers, and if the electric field lines cannot leave the channel because of a weakly polarising outside medium, then the relation $-\partial_x\overline{\Psi}(x) \propto 1/A(x)$ must hold. A fully analytical solution as in Eq. (S4) might not always be possible, however the inverse proportionality with the channel area $A(x)$ suffices for the qualitative mechanistic understanding discussed here. Consider, namely, a channel without any applied voltage such that the salt concentration $\overline{\rho}_{\rm s}(x,V)$ is constant within the channel. Upon applying a voltage, the electric field $-\partial_x\overline{\Psi}(x)$ will form quasi-instantaneously, so a short time after the voltage is applied we have a fully formed electric field, but still a constant salt concentration $\overline{\rho}_{\rm s}(x,V)$. In this case the diffusion term in Eq. (S10) vanishes and due to the aforementioned proportionality $-\partial_x\overline{\Psi}(x) \propto 1/A(x)$, the only x-dependence that remains in Eq. (S10) is $\overline{\rho_e}(x)$. Therefore, if we take the divergence $\partial_x J_x(x)$ of the total salt flux in Eq. (S10) and apply the continuity Eq. [S2] we find

$$\frac{\mathrm{d}\overline{\rho}_{\rm s}(x,V)}{\mathrm{d}t} = -\frac{\mathrm{d}J_x(x)}{\mathrm{d}x} \propto \overline{\rho_e}(x). \tag{S11}$$

This shows how any inhomogeneous ionic space charge density forms a source or sink for salt term upon applying a voltage, thereby inducing salt concentration polarisation and consequently current rectification. An important understanding is that the ionic charge *density* must be inhomogeneous, and not just the *total* ionic charge in the slab. The reason is that even though the total charge $Q_e(x) \equiv \overline{\rho_e}(x)A(x)$ in the slab for a constant space charge density $\overline{\rho_e}(x) = \overline{\rho_e}$ could still be x-dependent due to its scaling with $A(x)$, this dependence would cancel out with the $1/A(x)$ dependence of the electric field.

The insight that an inhomogeneous charge density forms a source-sink term previously explained how a constant surface charge density σ in a conical geometry could induce current rectification [2] as the total (surface) charge is in this case given by $Q_e(x) = 2\pi R(x)\sigma\mathrm{d}x$. Since in this geometry $A(x) = \pi R(x)^2$ we see that $Q_e(x) \propto \sqrt{A(x)}$ and therefore $\overline{\rho_e}(x) \equiv Q_e(x)/A(x) \propto 1/\sqrt{A(x)}$, exhibiting the required x-dependence. Additionally Eq. (S11) shows why merely a geometric inhomogeneity, such as a tapered geometry, is not enough to induce current rectification; it must go coupled with a spatially varying slab-averaged ionic charge density. The insight of Eq. (S11) could not only explain current rectification in channels with a space charge density step-function as in recent polyelectrolyte channels [12] and NCNM channels with colloids of opposing charge [11], it may also provide specific guidance to design current rectification properties in future iontronics.

1.4 Typical conductance memory retention time

As detailed in Refs. [3, 4], the process of ion accumulation and depletion is not instantaneous. To investigate this timescale for the channel of interest here we can apply the same approach as in Refs. [3, 4]. We consider two quantities, the total number of ions $N = \int_0^L 2R(x)H\varepsilon_{\rm fcc}\overline{\rho}_{\rm s}(x,V)\mathrm{d}x$ in the channel and the net salt flux $J_x(0) - J_x(L)$ into the channel. The change of N given by Eq. (S7) upon a small voltage perturbation V' around $V = 0$ yields

$$\begin{aligned}\left.\frac{\partial N}{\partial V}\right|_{V=0} V' =& \frac{\varepsilon_{\rm fcc} eHL\rho_{\rm e,t}\rho_{\rm e,b}\Delta RV'}{2k_{\rm B}T\ln^2\left(\frac{R_{\rm b}}{R_{\rm t}}\right)(\rho_{\rm e,t}R_{\rm t}-\rho_{\rm e,b}R_{\rm b})}\left((R_{\rm b}+R_{\rm t})\ln\left(\frac{\rho_{\rm e,t}}{\rho_{\rm e,b}}\right)\right.\\ &\left.+\frac{\ln\left(\frac{R_{\rm b}}{R_{\rm t}}\right)\left(-(\rho_{\rm e,t}-\rho_{\rm e,b})\Delta R(\rho_{\rm e,t}(R_{\rm b}+3R_{\rm t})-\rho_{\rm e,b}(3R_{\rm b}+R_{\rm t}))-2\ln\left(\frac{\rho_{\rm e,t}}{\rho_{\rm e,b}}\right)(\rho_{\rm e,t}R_{\rm t}-\rho_{\rm e,b}R_{\rm b})^2\right)}{(\rho_{\rm e,t}-\rho_{\rm e,b})^2\Delta R}\right)\\ \equiv&\alpha V',\end{aligned} \tag{S12}$$

where $\alpha > 0$ for our parameters, in agreement with the enhanced (reduced) conductance of a positive (negative) potential V' found in the experiments and as can be seen in Fig. 1(b).

At $V = 0$ the concentration profile is at equilibrium, so for a small voltage perturbation V' we can assume $\bar{\rho}_s(x) = 2\rho_b$. With this assumption the first term in Eq. (S5) vanishes. The net salt flux into the channel, $J_x(0) - J_x(L)$, is then determined by the remaining conductive terms

$$J_x(0) - J_x(L) = 2D\varepsilon_{\rm fcc}\frac{\Delta RH}{L\ln\left(\frac{R_{\rm b}}{R_{\rm t}}\right)}(\rho_{\rm e,t} - \rho_{\rm e,b})\frac{e}{k_{\rm B}T}V' \equiv \gamma V', \tag{S13}$$

where $\gamma > 0$ for our parameter choices, again in agreement with the enhanced (reduced) conductance of a positive (negative) potential V' found in the experiments and as can be seen in Fig. 1(b). We see that the net flux is determined by the difference in space charge density between the tip and the base $(\rho_{\rm e,t} - \rho_{\rm e,b})$. This is consistent with Eq. (S5), where we saw that the total salt flux is proportional to the charge density in the absence of a diffusion term. Therefore, this reconfirms the underlying mechanistic insight that a inhomogeneous charge density is what drives a net salt flux and consequent salt concentration polarisation, as also argued in Sec. 1 .3 .1.

The typical time it takes for ion depletion or accumulation, and thus the typical memory retention timescale, is then approximated by $\tau = \alpha/\gamma$. This yields

$$\begin{aligned}
\tau =& \frac{L^2\rho_{\rm e,t}\rho_{\rm e,b}\left((R_{\rm b}-R_{\rm t})(R_{\rm b}+R_{\rm t})\ln\left(\frac{\rho_{\rm e,t}}{\rho_{\rm e,b}}\right) + \frac{\ln\left(\frac{R_{\rm b}}{R_{\rm t}}\right)\left(-(\rho_{\rm e,t}-\rho_{\rm e,b})(R_{\rm b}-R_{\rm t})(\rho_{\rm e,t}(R_{\rm b}+3R_{\rm t})-\rho_{\rm e,b}(3R_{\rm b}+R_{\rm t}))-2\ln\left(\frac{\rho_{\rm e,t}}{\rho_{\rm e,b}}\right)(\rho_{\rm e,t}R_{\rm t}-\rho_{\rm e,b}R_{\rm b})^2\right)}{(\rho_{\rm e,t}-\rho_{\rm e,b})^2}\right)}{4D(\rho_{\rm e,t}-\rho_{\rm e,b})(R_{\rm b}-R_{\rm t})\ln\left(\frac{R_{\rm b}}{R_{\rm t}}\right)(\rho_{\rm e,t}R_{\rm t}-\rho_{\rm e,b}R_{\rm b})} \\
=& \frac{L^2}{4D}\rho_{\rm e,t}\rho_{\rm e,b}\left(\frac{(R_{\rm b}+R_{\rm t})\ln\left(\frac{\rho_{\rm e,t}}{\rho_{\rm e,b}}\right)}{(\rho_{\rm e,t}-\rho_{\rm e,b})\ln\left(\frac{R_{\rm b}}{R_{\rm t}}\right)(\rho_{\rm e,t}R_{\rm t}-\rho_{\rm e,b}R_{\rm b})} - \frac{\rho_{\rm e,t}R_{\rm b}-\rho_{\rm e,b}R_{\rm t}}{(\rho_{\rm e,t}-\rho_{\rm e,b})^2(\rho_{\rm e,t}R_{\rm t}-\rho_{\rm e,b}R_{\rm b})}\right. \\
&\left. - \frac{3}{(\rho_{\rm e,t}-\rho_{\rm e,b})^2} - \frac{2\ln\left(\frac{\rho_{\rm e,t}}{\rho_{\rm e,b}}\right)(\rho_{\rm e,t}R_{\rm t}-\rho_{\rm e,b}R_{\rm b})}{(\rho_{\rm e,t}-\rho_{\rm e,b})^3\Delta R}\right) \\
=& \frac{L^2}{4D}\left(\frac{(1+R_{\rm t}/R_{\rm b})\ln\left(\frac{\rho_{\rm e,t}}{\rho_{\rm e,b}}\right)}{(1-\rho_{\rm e,b}/\rho_{\rm e,t})\ln\left(\frac{R_{\rm b}}{R_{\rm t}}\right)(\rho_{\rm e,t}R_{\rm t}/(\rho_{\rm e,b}R_{\rm b})-1)} - \frac{1-\rho_{\rm e,b}R_{\rm t}/(R_{\rm b}\rho_{\rm e,t})}{(\rho_{\rm e,t}/\rho_{\rm e,b}+\rho_{\rm e,b}/\rho_{\rm e,t}-2)(R_{\rm t}/R_{\rm b}-\rho_{\rm e,b}/\rho_{\rm e,t})}\right. \\
&\left. - \frac{3}{\rho_{\rm e,t}/\rho_{\rm e,b}+\rho_{\rm e,b}/\rho_{\rm e,t}-2} - \frac{2\ln\left(\frac{\rho_{\rm e,t}}{\rho_{\rm e,b}}\right)(R_{\rm t}/R_{\rm b}-\rho_{\rm e,b}/\rho_{\rm e,t})}{(1-\rho_{\rm e,b}/\rho_{\rm e,t})(\rho_{\rm e,t}/\rho_{\rm e,b}+\rho_{\rm e,b}/\rho_{\rm e,t}-2)\Delta R/R_{\rm b}}\right).
\end{aligned}$$

From the above calculation it has now become clear that $\tau \propto L^2/(4D)$, which corresponds to the time it takes to diffuse over distances of the order of the channel length L. The remaining involved terms form a dimensionless number

$$\begin{aligned}
\xi =& \frac{(1+R_{\rm t}/R_{\rm b})\ln\left(\frac{\rho_{\rm e,t}}{\rho_{\rm e,b}}\right)}{(1-\rho_{\rm e,b}/\rho_{\rm e,t})\ln\left(\frac{R_{\rm b}}{R_{\rm t}}\right)(\rho_{\rm e,t}R_{\rm t}/(\rho_{\rm e,b}R_{\rm b})-1)} - \frac{1-\rho_{\rm e,b}R_{\rm t}/(R_{\rm b}\rho_{\rm e,t})}{(\rho_{\rm e,t}/\rho_{\rm e,b}+\rho_{\rm e,b}/\rho_{\rm e,t}-2)(R_{\rm t}/R_{\rm b}-\rho_{\rm e,b}/\rho_{\rm e,t})} \\
&- \frac{3}{\rho_{\rm e,t}/\rho_{\rm e,b}+\rho_{\rm e,b}/\rho_{\rm e,t}-2} - \frac{2\ln\left(\frac{\rho_{\rm e,t}}{\rho_{\rm e,b}}\right)(R_{\rm t}/R_{\rm b}-\rho_{\rm e,b}/\rho_{\rm e,t})}{(1-\rho_{\rm e,b}/\rho_{\rm e,t})(\rho_{\rm e,t}/\rho_{\rm e,b}+\rho_{\rm e,b}/\rho_{\rm e,t}-2)(1-R_{\rm t}/R_{\rm b})}
\end{aligned}$$

of order $\mathscr{O}(10^{-1})$ depending on the channel geometry $R_{\rm t}/R_{\rm b}$ and internal space charge distribution $\rho_{\rm e,b}/\rho_{\rm e,t}$. Fig. S2 shows the dependence of ξ for typical tip-to-base ratios of the space charge concentrations in Fig. S2(a) and the channel widths in Fig. S2(b). For our standard parameters we find $\xi \approx 0.42$. This simplifies our notation considerably such that we arrive at the final concise expression Eq. (S14) for τ which can be found in the main text as Eq. [1],

$$\tau = \frac{L^2}{4D}\xi. \tag{S14}$$

For our standard parameter set as laid out in Sec. 2, we have $\tau \approx 1.62$ s.

To then arrive at Eq. [2] of the main text, we make the natural assumption that the time-derivative of the dynamic conductance $\partial_t g(t)$ depends on the difference with the corresponding steady-state conductance, i.e. $\partial_t g(t) = f\big(g_\infty(V(t)) - g(t)\big)$ for some function f, where $f(0)$ must vanish based on stability arguments. By expanding f up to first order we find $\partial_t g(t) \propto g_\infty(V(t)) - g(t)$ with the proportionality constant naturally given by the typical timescale τ of the underlying salt concentration polarisation that drives the conductance change, given by Eq. (S14) (Eq. [1] in the main text).

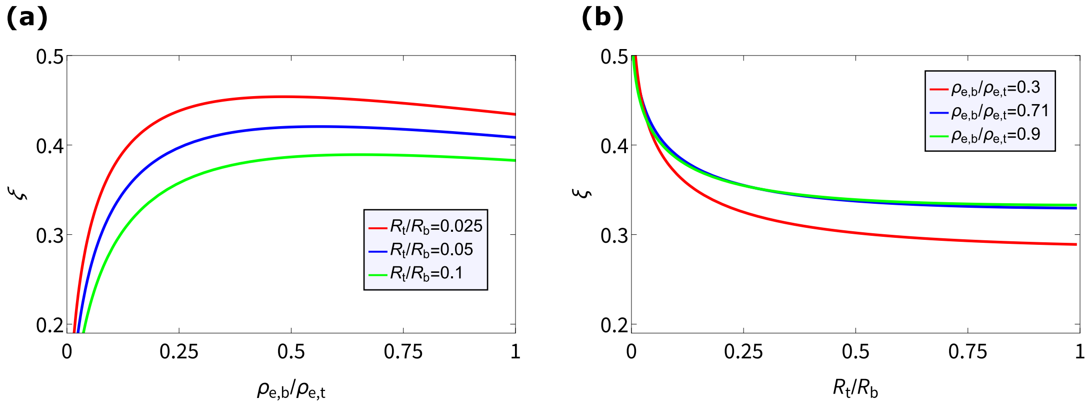

Fig. S2. The memory time proportionality function ξ as a function the internal space charge ratio $\rho_{\rm e,b}/\rho_{\rm e,t}$ in **(a)** and the tip and base radii ratio $R_{\rm t}/R_{\rm b}$ in **(b)**, for three different values of $R_{\rm t}/R_{\rm b}$ and $\rho_{\rm e,b}/\rho_{\rm e,t}$, respectively. In both **(a)** and **(b)** the blue graph represents $R_{\rm t}/R_{\rm b} = 0.05$ and $\rho_{\rm e,b}/\rho_{\rm e,t} = 0.71$, respectively, the system parameters used in the main text, except for Fig. 1(b) which uses $\rho_{\rm e,b}/\rho_{\rm e,t} = 0.90$.

1.5 Cell model

In Sec. 1 .2 we showed how the inhomogeneous charge distribution leads to salt concentration polarisation and thereby to ion current rectification. However, Eq. (S6) presented in Ref. [1], which describes the inhomogeneous space charge, follows from empirical measurements without a microscopic explanation of the actual origin of this important feature of the channel. Here we will leverage the well-established Poisson-Boltzmann cell-model [15, 16] to propose a tentative explanation of the emergence of the observed inhomogeneous space charge.

We consider a dispersion of charged colloidal spheres of radius a at packing fraction η in a 1:1 electrolyte of Debye length $\lambda_{\rm D}$, such that the volume per particle equals $(4\pi/3)b^3$ with $b \equiv a\eta^{-1/3} > a$. The environment of each particle fluctuates due to colloidal Brownian motion, and hence the calculation of the profile of the electric potential $\psi(\mathbf{r};\{\mathbf{R}\})$ is a complicated many-body problem that depends on the instantaneous configuration $\{\mathbf{R}\}$ of the colloidal particles. This problem can be reduced tremendously, however, if we assume each sphere to be at the center of an electrically neutral and spherically symmetric Wigner-Seitz cell of radius b. The dimensionless electrostatic potential $\phi(r) \equiv e\psi(r)/k_{\rm B}T$ is then the same in each cell, and can for $r \in [a,b]$ be described by the Poisson-Boltzmann equation with boundary conditions

$$\phi''(r) + \frac{2}{r}\phi'(r) = \lambda_{\rm D}^{-2}\sinh\phi(r), \tag{S15}$$

$$\phi'(b) = 0, \tag{S16}$$

$$\phi(a) = \phi_0, \tag{S17}$$

where a prime denotes a radial derivative and where ϕ_0 is the fixed dimensionless zeta potential in units of $k_{\rm B}T/e \simeq 25$ mV. This is a closed system of equations that can easily be solved numerically for fixed a, b, $\lambda_{\rm D}$, and ψ_0 or rather for fixed dimensionless η, $a/\lambda_{\rm D}$ and ϕ_0. The resulting surface charge density $e\sigma$ of the spheres is then from Gauss's law given by $\sigma = -\phi'(a)/4\pi\lambda_B$ with $\lambda_{\rm B} = e^2/4\pi\varepsilon k_{\rm B}T = 0.72$ nm the Bjerrum length of water.

For dispersions that are dilute enough that $b - a \gg \lambda_{\rm D}$, the EDLs described by Eqs.(S15) are fully developed. In this regime it is well known that the colloidal particles obtain their maximum (absolute) surface charge [17]. At high packing fraction where $b - a \simeq \lambda_{\rm D}$, the colloidal surfaces discharge and loose their charge completely in the (unphysical) limit $b = a$. The situation for the colloidal dispersion of our system is intricate, since for $a = 100$ nm and $\eta = 0.74$ we have $b/a \simeq 1.105$ and hence $b - a \simeq 3\lambda_{\rm D}$. In this regime the assumption of spherical symmetry of the EDLs is highly questionable, because the surface-surface distances between a central particle in a close-packed fcc crystal at $\eta = 0.74$ vary between smaller than $\lambda_{\rm D}$ in the 12 directions of its nearest neighbours to about $10\lambda_{\rm D}$ in the directions of the 8 tetrahedral and the 6 octahedral holes of the fcc crystal. We can

therefore expect that the particles only significantly discharge in the vicinity of nearest-neigbour contact. Within the spherical-cell approximation, we mimic this anisotropy of the colloidal surface charge by averaging over these $12+8+6=26$ directions, with equal weight for simplicity, to define the effective surface charge density $\sigma^* = (12\sigma_2 + 14\sigma_\infty)/26$, with the surface charges σ_2 and σ_∞ of the spherical cell with b half the nearest-neighbour distance and $b \gg a$ the dilute limit, respectively. In Fig. S3 we show the dependence of σ^*/σ_∞ on the packing fraction η for the present system parameter with $\phi_0 = -1.53$, which is such that $\sigma_C = \sigma_\infty = 0.01$ C/m^2. It shows that decreasing the packing fraction from $\eta = 0.74$ to 0.73, which corresponds to reducing b by as little as 0.5 nm (i.e. by $\simeq \lambda_D/6$), the colloidal charge can increase by as much as $\sim 20\%$. A reduction to $\eta \simeq 0.67$ and $\eta \simeq 0.63$ (which corresponds to $b-a=\lambda_D$ and $2\lambda_D$) increases the surface charge by about 80% and 100%, respectively, compared to that at $\eta = 0.74$. On the basis of these simple estimates, we argue that the slab-averaged space charge in the channel (that compensates the colloidal surface charge) can therefore also exhibit the same relative change between tip and base. The variation we use of $\rho_{e,b}/\rho_{e,t} = 0.90$ for the steady-state results of Fig. 1(b) and $\rho_{e,b}/\rho_{e,t} = 0.71$ for the other (dynamic) results falls well within this reasonable range of change predicted by the explanation we offer here. We leave an additional estimate on an alleged spatial variation of ϕ_0 in the channel to explain the heterogeneous space charge as future work.

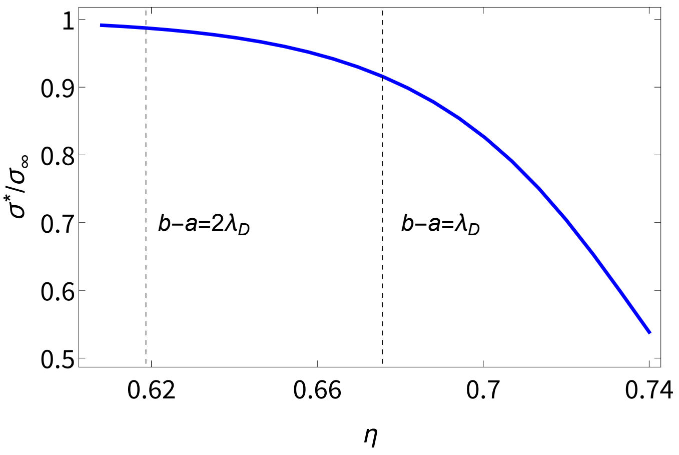

Fig. S3. Dependence on the colloidal packing fraction η of the effective surface charge density σ^* of a colloid in an fcc crystal as a fraction of the surface charge density $\sigma_\infty = \sigma_C = 0.01$ C/m^2 of a free isolated colloid. An increase of up to $\sim 100\%$ in σ^* is already visible when the cell radius b in the direction of the nearest neighbour just extends one or two Debye lengths λ_D beyond the colloid of radius a, i.e. when the nearest neighbour distance $b-a \in [0, 2\lambda_D]$.

2 System parameters

The channel has a base radius of $R_b = 100\mu$m, a tip radius of $R_t = 5\mu$m and a height of $H = 5\mu$m. The channel connects two reservoirs with a bulk concentration $\rho_b = 10$ mM of aqueous KCl electrolyte, yielding a Debye length of $\lambda_D \approx 3.1$ nm. The colloids have a radius of $a = 100$ nm and carry a uniform charge density of $\sigma_C = -0.01$ C/m^2, squeezed together during the device fabrication to form a face centered cubic crystal with a close-packed packing fraction $\eta = 1 - \varepsilon_{fcc} \approx 0.74$, where ε_{fcc} is the porosity. The maximum space charge at the tip is determined in the same way as in Ref. [1], i.e. $\overline{\rho_e}(L) = \rho_{e,t} = 4\pi a^2 \sigma_C n/(e\varepsilon_{fcc}\Omega) = 8.85$ mM with $n = \frac{3}{4}\eta\Omega/(\pi a^3)$ the number of colloids in the channel and Ω the overall channel volume. The space charge concentration at the base is assumed to be $\overline{\rho_e}(0) = \rho_{e,b} = 8$ mM in the steady-state calculations for Fig. 1(b) and $\rho_{e,b} = 6.3$ mM in the rest of the manuscript for the time-dependent calculations. These values were chosen such that (i) the change in space charge density does not exceed the reasonable regime predicted by our tentative explanation for the the inhomogeneous space charge in Sec. 1 .5, i.e. the difference between $\rho_{e,b}$ and $\rho_{e,b}$ is not more than $\sim 40\%$, and (ii) a good agreement is found with the experiments. Although the space charge density $\overline{\rho_e}(x)$ of Eq. (S6) has a clear physical meaning, its precise form and parameters values are not entirely clear and may contain a voltage-dependence that we ignore in the present study and leave for future investigations. We stress, however, that our present overall results do not depend strongly on the detailed value of $\rho_{e,b}$ and $\rho_{e,t}$, as it only marginally changes the conductance properties in the parameter regime we consider here and the overall behaviour relevant to reservoir computing remains. However, we do note that our theory predicts that some difference between the space charge density at the tip and at the base is necessary for any current rectification, so even though the precise values of $\rho_{e,b}$ and $\rho_{e,t}$ are not of major importance for our overall results, our theory still predicts it is crucial that there is some difference between the two. Lastly, the effective diffusion coefficient is also taken to be similar to Ref. [1] with $D_{eff} = \varepsilon_{fcc} D = 0.38$ μm^{2}ms^{-1}.

3 Device fabrication

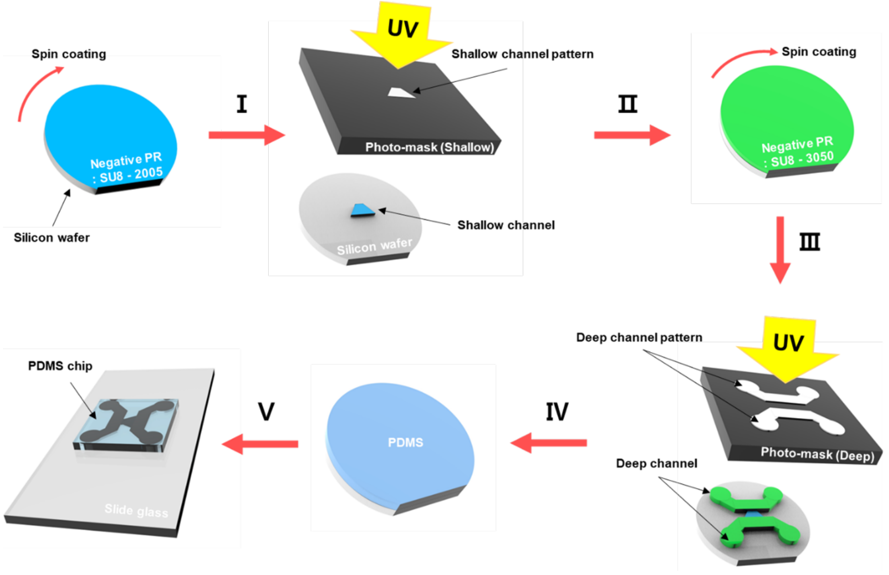

Fig. S4. Soft lithography process to construct our microfluidic memristor.

Fig. S4 shows schematic diagrams describing the fabrication procedures for microfluidic devices using soft lithography: A negative photoresist (PR) was applied to the silicon wafer (SU-8 2005; Microchem Co., Westborough, Massachusetts, USA) using a spin coater and then soft-baked. The PR was then exposed to UV to create a shallow channel and the wafer was hard baked. The unexposed PR was removed to form the shallow 5 μm channel. A deep 100 μm channel was then formed using a negative photoresist, SU8-3050 (Microchem Co., Westborough, Massachusetts, USA), in the same process as above. After completion of the master mold, the surface was treated with (3,3,3-trifluoropropyl)silane (452807; Sigma-Aldrich, St. Louis, Missouri, USA). Polydimethylsiloxane (PDMS; Sylgard; Dow Corning Korea Ltd., Gwangju-si, Gyeonggi-do, Republic of Korea) was then poured over the master mold and heated on a hot plate at 95 °C for 1 hour. The reservoir of the PDMS device was punched out with a 1.5 mm medical punch. The surface of the PDMS and the slide glass were treated using a plasma device (Cute-MP; Femto Science, Hwaseong-si, Gyeonggi-do, Republic of Korea) and bonded together.

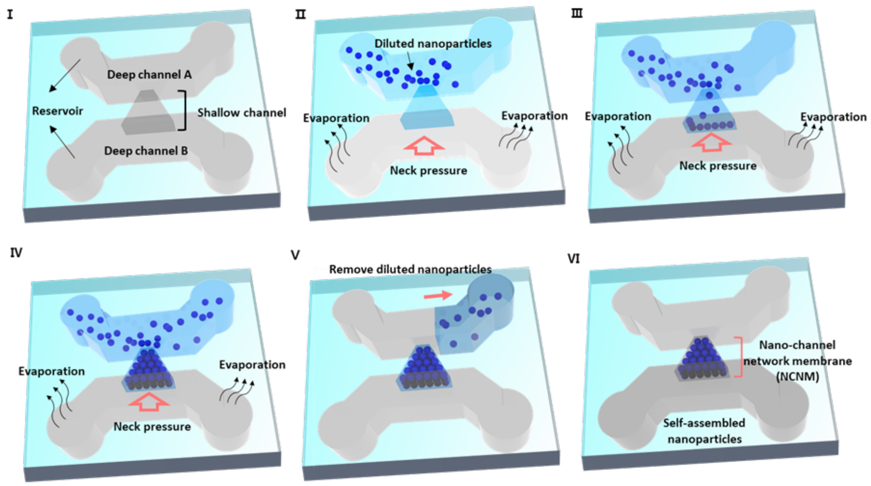

Fig. S5. Schematic depiction of in situ nanoparticle assembly in the microchannel.

As shown in Fig. S5, the diluted nanoparticles (with a carboxylic (-COOH) end groups on the surface and with radius $a =$ 100 nm) dispersed in a 2 μL 70% ethanol solution were injected into the deep channel, such that the solution including the nanoparticles fills the shallow channel through the capillary. Due to the neck pressure at the interface between the shallow

and deep channels, the flow of the particle dispersion stopped, after which evaporation of the solvent (70% of the ethanol) was induced through the deep channel. This way additional nanoparticles were transported toward the neck by the convective flow that compensates for the loss of solvent by evaporation. This influx of particles promotes the growth (and the compression) of the ordered fcc lattice in the shallow channel. When the self-assembled nanoparticles completely filled the shallow channel with a wet close-packed fcc lattice, the remaining diluted nanoparticles were removed from the deep channel by suction. The finished device was dried for one day and then used for the experiment.

4 Additional experimental results

4.1 Voltage pulse measurements

To obtain Fig. 4(a) we applied all $2^4 = 16$ different voltage trains of four pulses in sequence with 30 s between each pulse train. This was performed on three different devices with two cycles per device, resulting in 6 independent measurements per voltage train, all individually shown in Fig. S6. The average of these measurement is shown in Fig. 4(a), the standard deviations calculated with the six conductance values after the fourth (last) pulse for each pulse train are shown in Table I. These values are used in the main text to take the measured (device-to-device) variability into account. Therefore we stress that all (device-to-device) variability visible in Fig. S6 is incorporated in the main text.

To ensure that our devices also remain reliable and stable over longer periods of time, we performed various additional cycles of pulse trains over a single device. In Fig. S7 we show a 50 cycle repeat, which lasted about 30 minutes, of the pulse train corresponding to the bitstring 0101. We see a remarkable stability, with essentially the same current response each cycle (which we also show as Fig. 2 in the main text), yielding a narrow spread for each pulse and reliably measured altered conductances with conductance standard deviations of $g/g_0 \sim 0.03 - 0.05$. Moreover, we cycled through all 16 different bitstrings for a total of 26 cycles, lasting around 4 hours, and found essentially the same current response for each cycle, even after the device had been cycling for 4 hours. The resulting average normalized conductances per pulse, per cycle, are shown in Fig. S8, with the corresponding standard deviations in the range $g/g_0 \sim 0.02 - 0.15$.

We repeated a similar protocol, but now with the ground at the base and the applied voltage at the channel tip. In this instance, a "0" corresponds to a pulse of 2 V, while a "1" corresponds to a pulse of -5 V. Pulse duration and interval are still 0.75 s, the read pulses are 1 V of 50 ms duration. The result is shown in Fig. S9, showing a similar clear separation of the different bit-strings. To again ensure the device stability, the bit-strings 1111, 0101 and 0011 were repeated 5 times, which we present in Fig. S10. Here we show the 5 individual measurements (light grey), the average of the measured current (black) and the calculated normalized conductances in the bar plots, averaged over the 5 measurements. The error bars depict the measured standard deviations, where we find good reproducibility for each voltage pulse train.

In Fig. S11 we schematically show how we use the read pulses to calculate the channel conductance. We calculate the difference in (average) current during a read pulse and the measured current just before the read pulse to calculate the conductance.

In the main text we classified simple single-digit images consisting of 4×5 black and white pixels. In Fig. 3(b) we only showed the "2" as an example, the other digits used are depicted in Fig. S12.

4.2 Timescale measurements

In Fig. 2 we show measured hysteresis loops for 3 different frequencies for channels of lengths 50 μm, 100 μm and 150 μm. Additional measurements were conducted for intermediate frequencies in order to find upper and lower bound for the frequency which exhibits the most open hysteresis loop. All loops are shown in Fig. S13, Fig. S14 and Fig. S15 for channel lengths of 50 μm, 100 μm and 150 μm respectively.

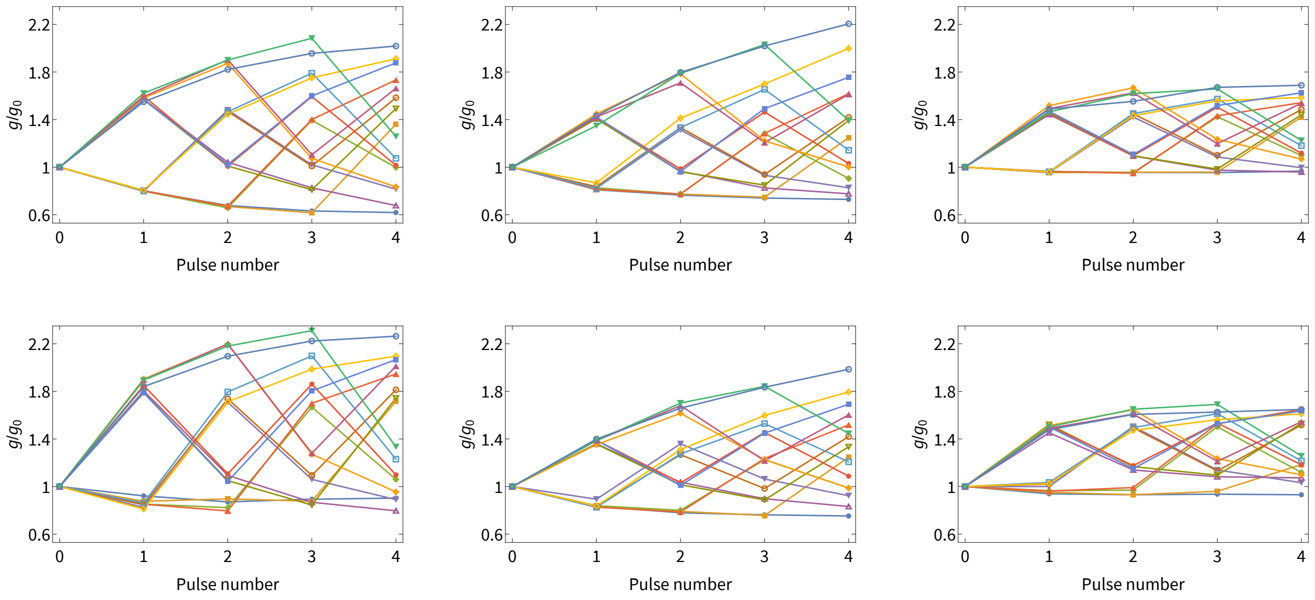

Fig. S6. Six measurements of the normalized channel conductance for all $2^4 = 16$ different voltage pulse trains, obtained from three devices. Each column of two figures corresponds to a device. The average for each different pulse train is used to create Fig. 4(a). Standard deviations for the conductance measurement after the fourth (last) pulse were also calculated with these six measurements and shown in Table I.

**Bit-string**	**Standard deviation**	**Bit-string**	**Standard deviation**
0000	0.137	1000	0.142
0001	0.193	1001	0.143
0010	0.080	1010	0.061
0011	0.157	1011	0.170
0100	0.088	1100	0.093
0101	0.148	1101	0.178
0110	0.058	1110	0.087
0111	0.207	1111	0.256

TABLE I. The standard deviations of the normalized channel conductance g/g_0 for each different bit-string, determined through the six measurements per bit-string shown in Fig. S6.

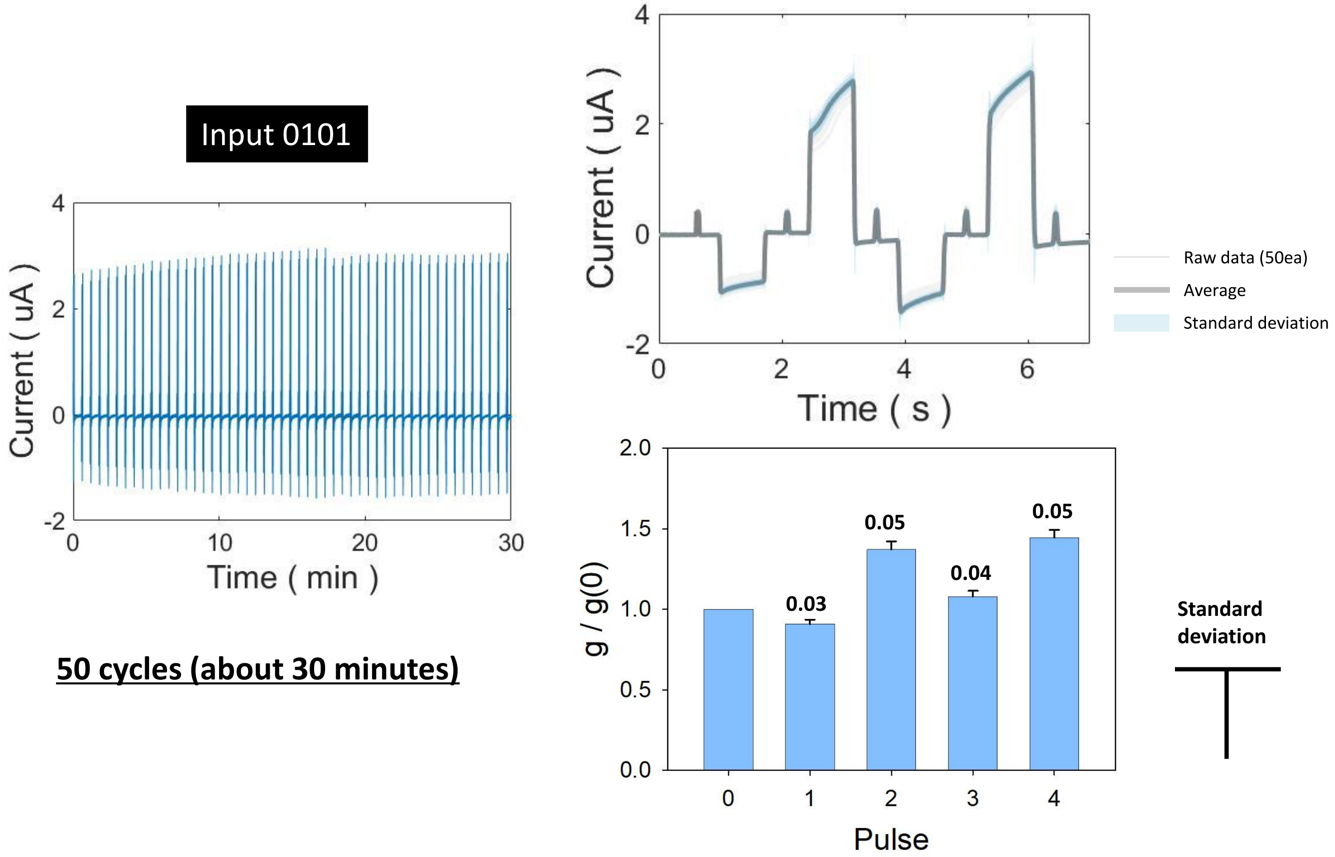

Fig. S7. A 50-cycle repeat of the 0101 voltage pulse train. With (left) the current measurement during all cycles, (top right) all cycles overlaid with the light grey spread the raw overlaid data, the dark grey line the average of the measurements, and the variability characterised by the standard deviation in light blue. (bottom right) The resulting normalized conductances determined by the five read-pulses with their respective standard deviations.

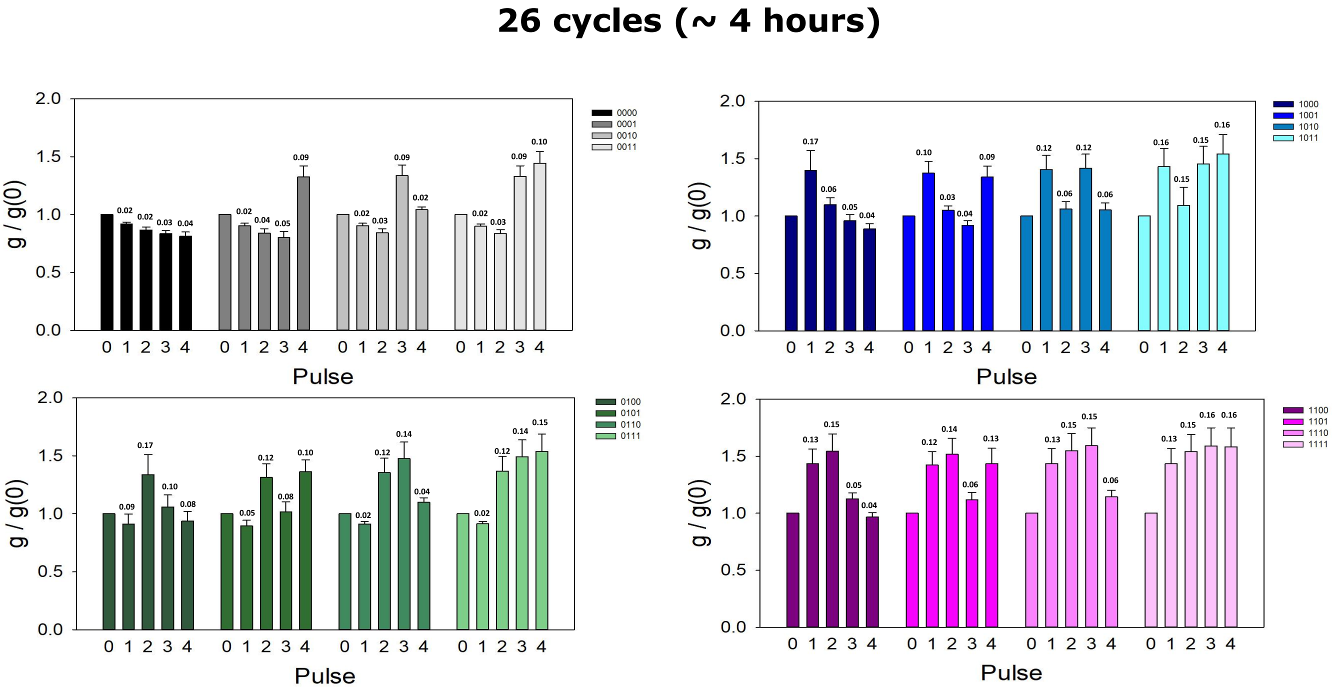

Fig. S8. Normalized conductances and their respective standard deviations obtained by cycling all 16 voltage pulse trains for a total of 50 times, taking roughly 4 hours to complete.

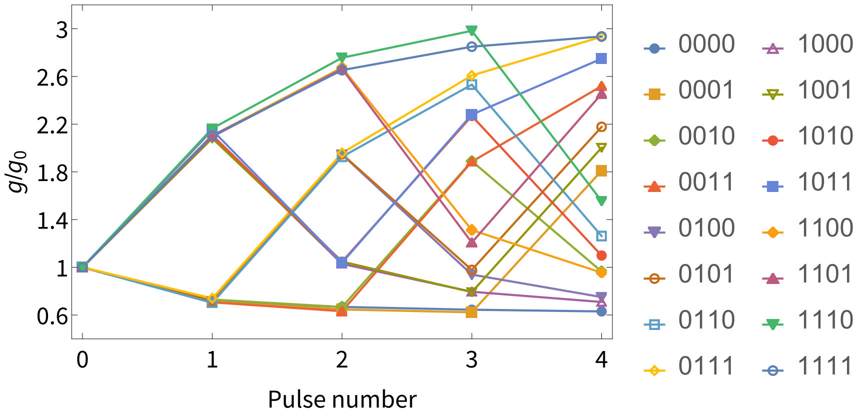

Fig. S9. Measurements of the normalized channel conductance for all $2^4 = 16$ different voltage pulse trains, but here the channel base is grounded and the voltage is applied at the tip.

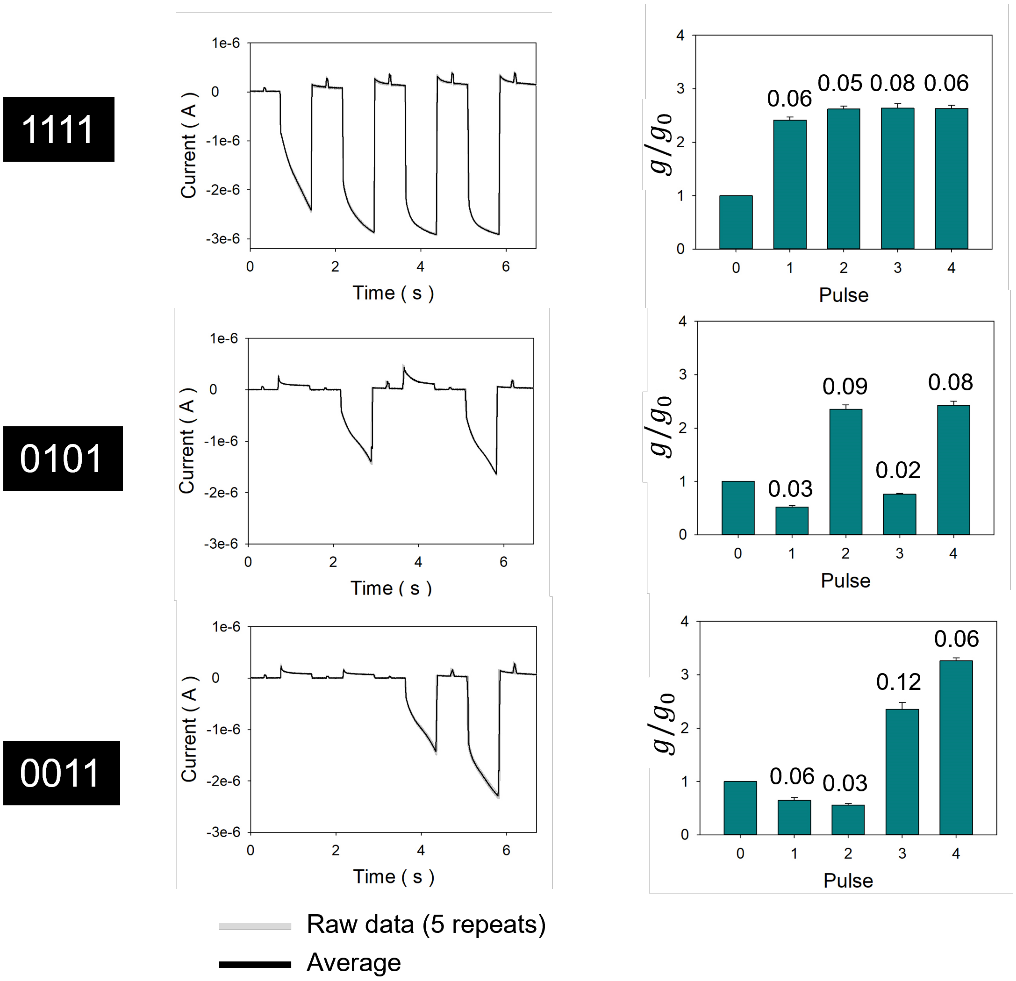

Fig. S10. Voltage pulse train measurements with the ground at the tip and the applied voltage at the channel base. A “0" corresponds to a pulse of 2 V, while a “1" corresponds to a pulse of -5 V. Pulse duration and interval are 0.75 s, with read pulses of 1 V and 50 ms duration. Voltage pulse trains corresponding to the bit-strings 1111, 0101 and 0011 were repeated 5 times, where we show the 5 individual measurements (light grey), the average of the measured current (black) and the calculated normalized conductances in the bar plots, averaged over the 5 measurements. The error bars depict the measured standard deviations.

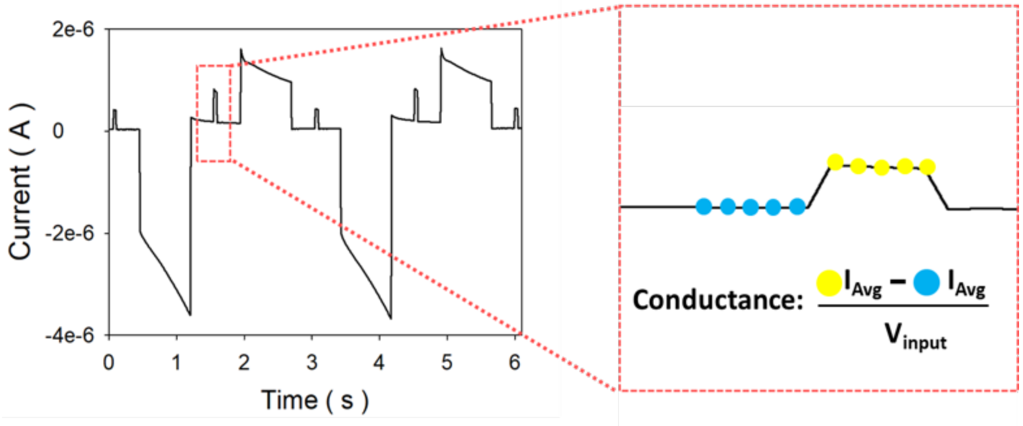

Fig. S11. Schematic depiction of how the read-pulses are used to calculate the channel conductance. The current measurements during the read pulse are averaged. The difference with the average current just before the read pulse then yields the channel conductance after dividing by the applied voltage. Before each voltage pulse train, a read pulse is applied to obtain the base conductance g_0, which is used to normalise the measurements.

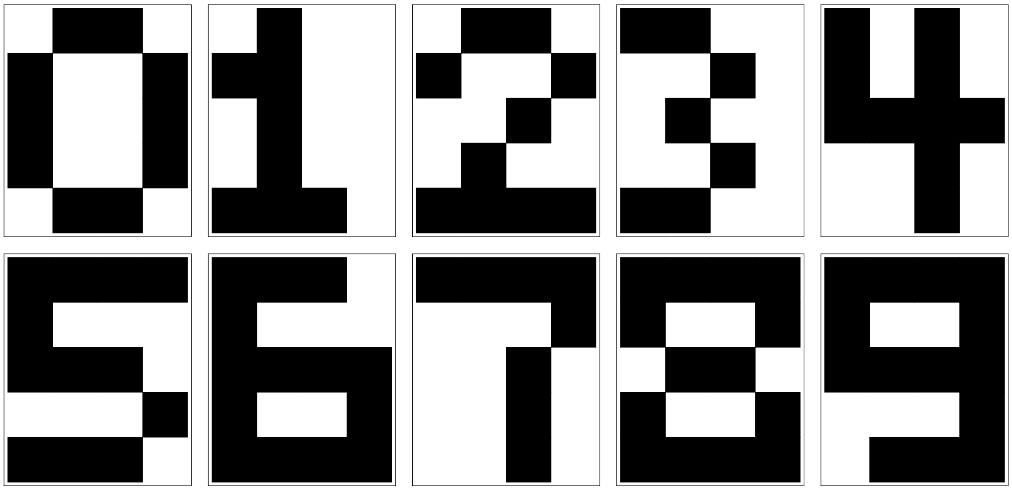

Fig. S12. Simple single digit numbers used for classification in the main text as shown in Fig. 3(b) and Fig. 3(c).

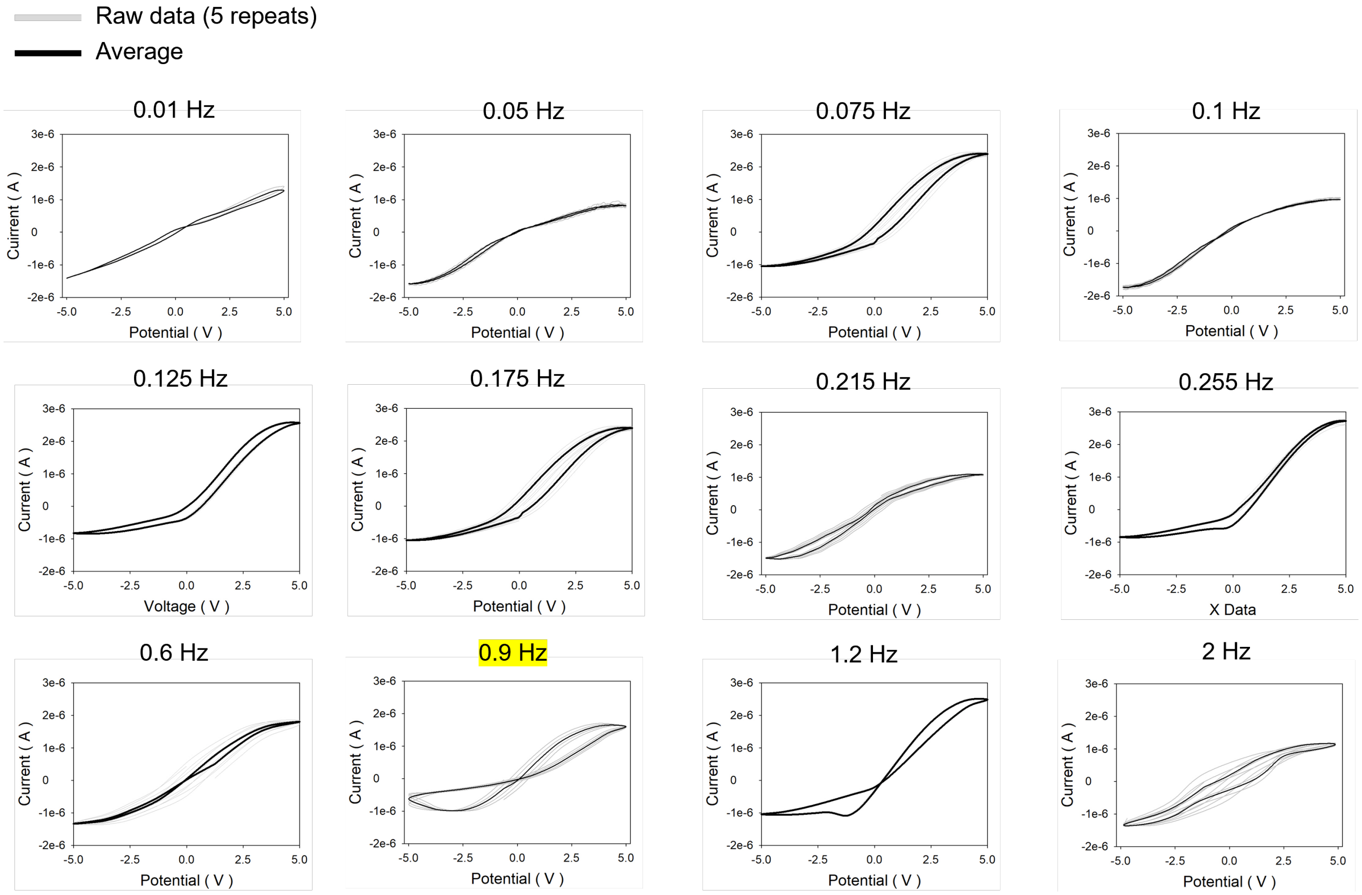

Fig. S13. Current-voltage hysteresis loops for the 50 μm channel as a result of a sinusoidal voltage over the channel of amplitude 5 V for the various frequencies shown. Measurements were gathered during five periods, depicted as the light grey graphs, where the average of the measurements is shown as a black graph. Enclosed areas were calculated with $f_{\mathrm{max}} = 0.9$ Hz (highlighted yellow) exhibiting the most open hysteresis loop.

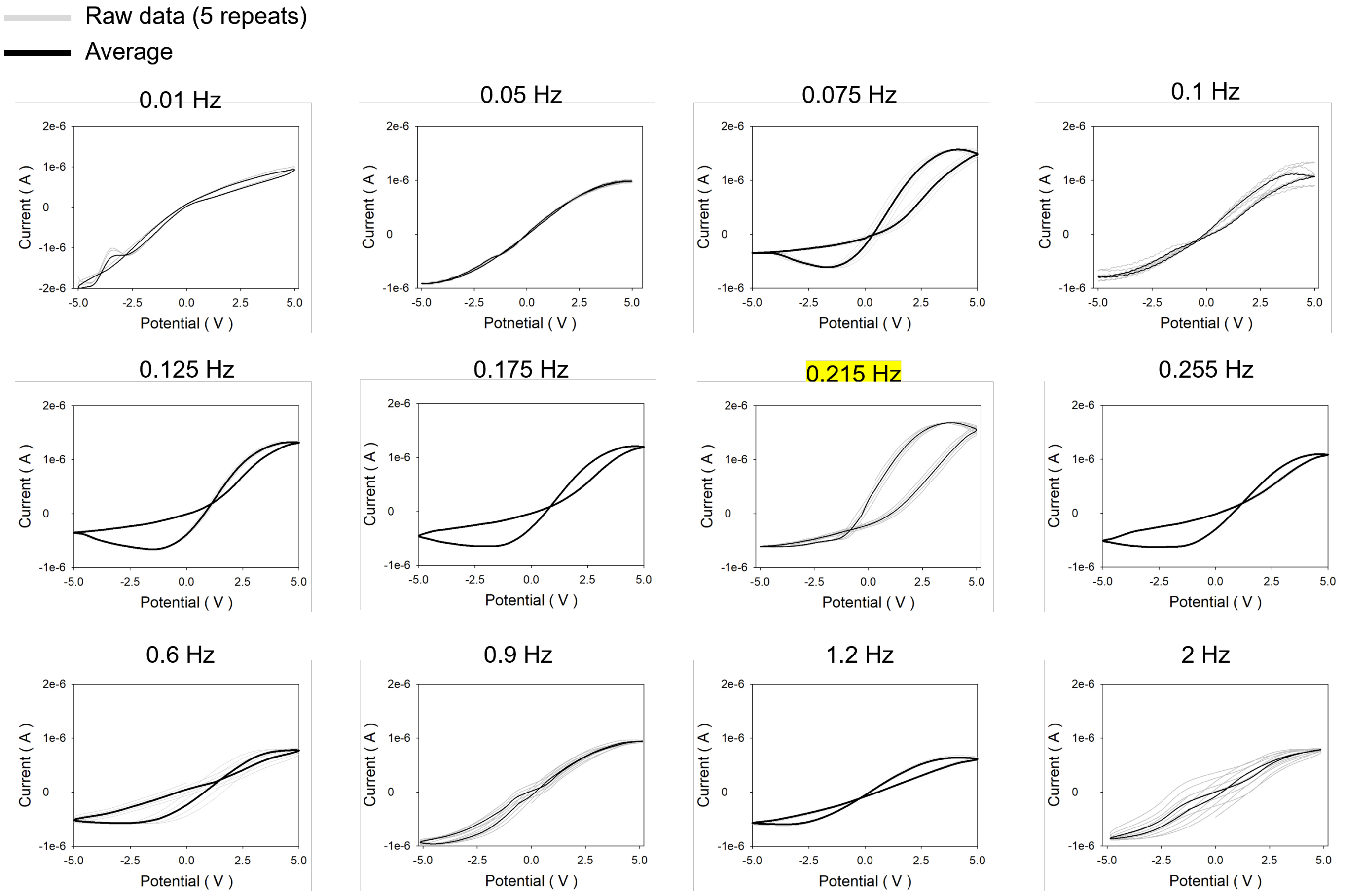

Fig. S14. Current-voltage hysteresis loops for the 100 μm channel as a result of a sinusoidal voltage over the channel of amplitude 5 V for the various frequencies shown. Measurements were gathered during five periods, depicted as the light grey graphs, where the average of the measurements is shown as a black graph. Enclosed areas were calculated with $f_{\mathrm{max}} = 0.215$ Hz (highlighted yellow) exhibiting the most open hysteresis loop.

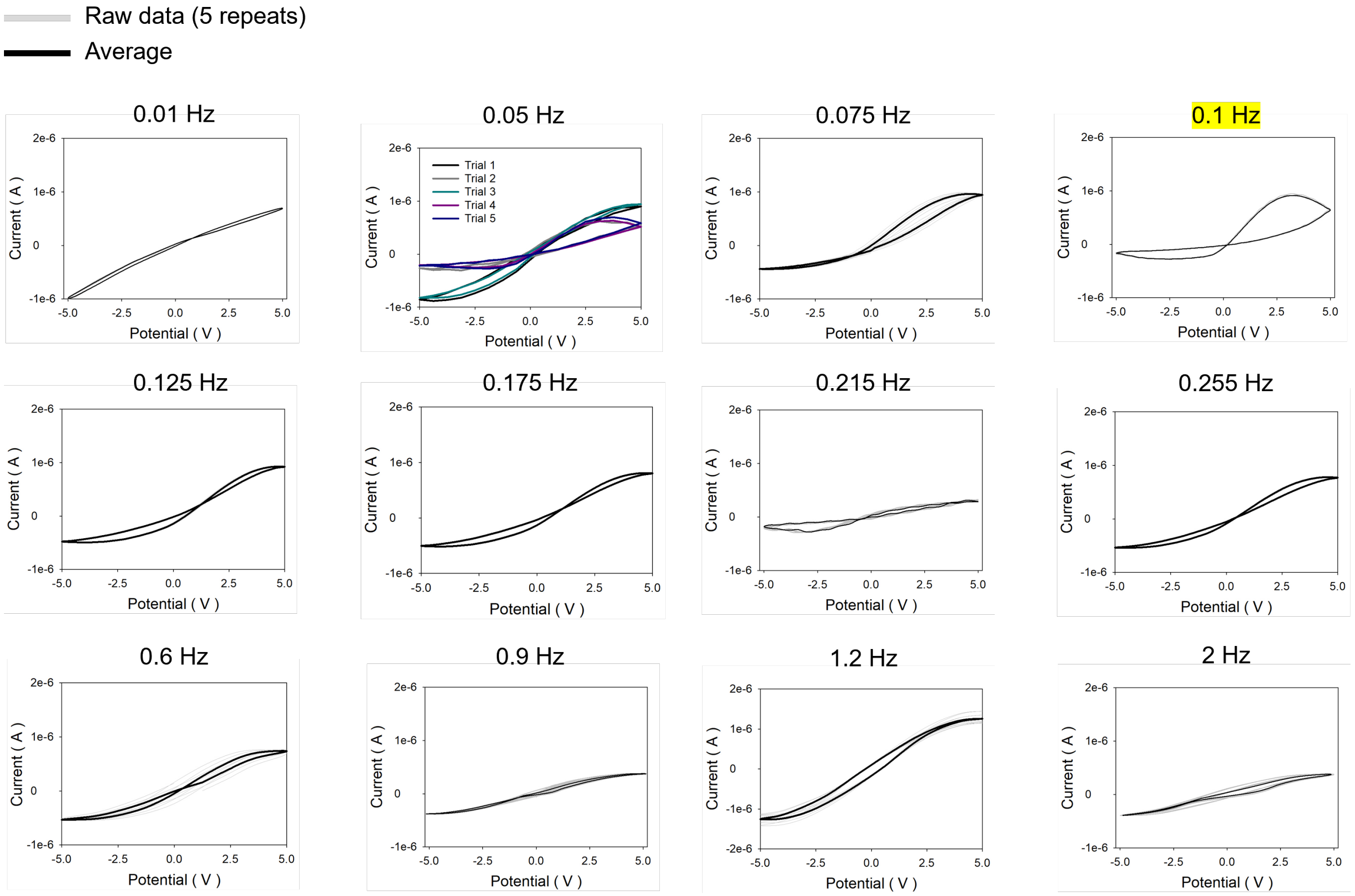

Fig. S15. Current-voltage hysteresis loops for the 150 μm channel as a result of a sinusoidal voltage over the channel of amplitude 5 V for the various frequencies shown. Measurements were gathered during five periods, depicted as the light grey graphs, where the average of the measurements is shown as a black graph. Enclosed areas were calculated with $f_{\text{max}} = 0.1$ Hz (highlighted yellow) exhibiting the most open hysteresis loop. In the first run of measurements, 0.05 Hz yielded a hysteresis loop with an enclosed area very close to 0.1 Hz. Both frequencies have been tested various more times, where the 0.1 Hz loops were consistently more open, one of these experiments was used for Fig. 1(c). The other 0.05 Hz measurements are shown here.

[1] E. Choi, C. Wang, G. T. Chang, and J. Park, High current ionic diode using homogeneously charged asymmetric nanochannel network membrane, Nano Letters **16**, 2189 (2016).
[2] W. Q. Boon, T. E. Veenstra, M. Dijkstra, and R. van Roij, Pressure-sensitive ion conduction in a conical channel: optimal pressure and geometry, Physics of Fluids **34**, 101701 (2022).
[3] T. M. Kamsma, W. Q. Boon, T. ter Rele, C. Spitoni, and R. van Roij, Iontronic neuromorphic signaling with conical microfluidic memristors, Phys. Rev. Lett. **130**, 268401 (2023).
[4] T. M. Kamsma, W. Q. Boon, C. Spitoni, and R. van Roij, Unveiling the capabilities of bipolar conical channels in neuromorphic iontronics, Faraday Discussions (2023).
[5] O. Soloveva, S. Solovev, R. Zaripova, F. Khamidullina, and M. Tyurina, Evaluation of the effective porosity of an open cell foam material for using in heat and mass transfer numerical simulations, in *E3S Web of Conferences*, Vol. 258 (EDP Sciences, 2021) p. 11010.
[6] T. Heidig, T. Zeiser, and H. Freund, Influence of resolution of rasterized geometries on porosity and specific surface area exemplified for model geometries of porous media, Transport in Porous Media **120**, 207 (2017).
[7] A. Mani, T. A. Zangle, and J. G. Santiago, On the propagation of concentration polarization from microchannel- nanochannel interfaces part i: analytical model and characteristic analysis, Langmuir **25**, 3898 (2009).
[8] T. A. Zangle, A. Mani, and J. G. Santiago, On the propagation of concentration polarization from microchannel- nanochannel interfaces part ii: numerical and experimental study, Langmuir **25**, 3909 (2009).
[9] A. Mani and M. Z. Bazant, Deionization shocks in microstructures, Physical Review E **84**, 061504 (2011).
[10] M. Aarts, W. Q. Boon, B. Cuénod, M. Dijkstra, R. van Roij, and E. Alarcon-Llado, Ion current rectification and long-range interference in conical silicon micropores, ACS Applied Materials & Interfaces (2022).
[11] J. Kim, J. Jeon, C. Wang, G. T. Chang, and J. Park, Asymmetric nanochannel network-based bipolar ionic diode for enhanced heavy metal ion detection, ACS nano **16**, 8253 (2022).
[12] B. Sabbagh, N. E. Fraiman, A. Fish, and G. Yossifon, Designing with iontronic logic gates-from a single polyelectrolyte diode to an integrated ionic circuit, ACS Applied Materials & Interfaces **15**, 23361 (2023), pMID: 37068481, https://doi.org/10.1021/acsami.3c00062.
[13] P. Robin, N. Kavokine, and L. Bocquet, Modeling of emergent memory and voltage spiking in ionic transport through angstrom-scale slits, Science **373**, 687 (2021).
[14] P. Robin, T. Emmerich, A. Ismail, A. Niguès, Y. You, G.-H. Nam, A. Keerthi, A. Siria, A. Geim, B. Radha, *et al.*, Long-term memory and synapse-like dynamics in two-dimensional nanofluidic channels, Science **379**, 161 (2023).
[15] H. Von Grünberg, R. van Roij, and G. Klein, Gas-liquid phase coexistence in colloidal suspensions?, Europhysics Letters **55**, 580 (2001).
[16] E. Trizac, L. Bocquet, M. Aubouy, and H.-H. von Grünberg, Alexander's prescription for colloidal charge renormalization, Langmuir **19**, 4027 (2003).
[17] F. Smallenburg, N. Boon, M. Kater, M. Dijkstra, and R. van Roij, Phase diagrams of colloidal spheres with a constant zeta-potential, The Journal of chemical physics **134** (2011).
